# MiRNA-146a/AKT/β-Catenin Activation Regulates Cancer Stem Cell Phenotype in Oral Squamous Cell Carcinoma by Targeting CD24

**DOI:** 10.3389/fonc.2021.651692

**Published:** 2021-10-12

**Authors:** Sangeeta Ghuwalewala, Dishari Ghatak, Sumit Das, Stuti Roy, Pijush Das, Ramesh Butti, Mahadeo Gorain, Somsubhra Nath, Gopal C. Kundu, Susanta Roychoudhury

**Affiliations:** ^1^ Cancer Biology and Inflammatory Disorder Division, Council of Scientific and Industrial Research (CSIR)-Indian Institute of Chemical Biology, Kolkata, India; ^2^ Laboratory of Tumor Biology, Angiogenesis and Nanomedicine Research, National Centre for Cell Science (NCCS), Pune, India; ^3^ Division of Research, Saroj Gupta Cancer Centre and Research Institute, Kolkata, India

**Keywords:** mir-146a, β-catenin, Wnt-AKT signalling, CD24, stemness, OSCC

## Abstract

CD44^high^CD24^low^ population has been previously reported as cancer stem cells (CSCs) in Oral Squamous Cell Carcinoma (OSCC). Increasing evidence suggests potential involvement of microRNA (miRNA) network in modulation of CSC properties. MiRNAs have thus emerged as crucial players in tumor development and maintenance. However, their role in maintenance of OSCC stem cells remains unclear. Here we report an elevated expression of miR-146a in the CD44^high^CD24^low^ population within OSCC cells and primary HNSCC tumors. Moreover, over-expression of miR-146a results in enhanced stemness phenotype by augmenting the CD44^high^CD24^low^ population. We demonstrate that miR-146a stabilizes β-catenin with concomitant loss of E-cadherin and CD24. Interestingly, CD24 is identified as a novel functional target of miR-146a and ectopic expression of CD24 abrogates miR-146a driven potential CSC phenotype. Mechanistic analysis reveals that higher CD24 levels inhibit AKT phosphorylation leading to β-catenin degradation. Using stably expressing miR-146a/CD24 OSCC cell lines, we also validate that the miR-146a/CD24/AKT loop significantly alters tumorigenic ability *in vivo*. Furthermore, we confirmed that β-catenin trans-activates miR-146a, thereby forming a positive feedback loop contributing to stem cell maintenance. Collectively, our study demonstrates that miR-146a regulates CSCs in OSCC through CD24-AKT-β-catenin axis.

## Introduction

OSCC is the most prevalent form of head and neck cancers worldwide with more than 60% individuals diagnosed with advanced tumors (1). The oral CSCs are held responsible for tumor aggressiveness leading to treatment failures, relapse and development of metastases (2, 3). Over the past decade, epigenetic re-programming has emerged as a crucial mechanism of regulating cancer stem cell dynamics (4, 5). These include DNA methylation, histone modifications and chromatin remodeling having robust effect upon cellular fate and stem cell potential. Another such epigenetic regulatory mechanism that has recently gained considerable importance in tumor biology are the miRNAs. MiRNAs are small ncRNAs of 20-22 nucleotides, de-regulation of which may have critical roles in disease development. They can act either as oncogenes or as tumor-suppressors depending upon the specific genes targeted (6, 7). MiRNA associated signatures are now considered for cancer specific diagnostic and prognostic purposes (8, 9).

MiRNAs not only regulate primary cellular functions like proliferation, differentiation, migration and invasion, but also directly or indirectly influence CSC functions (10). These are mostly attributed to altered signaling pathways including cell surface markers, pluripotency factors, chemo-resistance and epithelial-to-mesenchymal transition (EMT) markers (11–14). Role of miR-34a, miR-145a and miR-200bc family in regulating CD44, Oct4, Sox2, KLF4, Bmi1, Zeb1/2 and Notch1 has been well established (15–17). Thus, by precisely regulating the CSC related genes, miRNAs themselves have emerged as inherent modulators of cancer stem cells. MiR-146a is predominantly an onco-miR, which directly targets *IRAK1*, *traf6*, and *numb* genes in OSCC and imparts tumorigenicity (18, 19). Emerging evidence on miR-146a suggests that it directs the self-renewal process in colorectal cancer stem cells by regulating Snail-β-catenin axis which also contributes to EMT (20). High nuclear accumulation of β-catenin, along with lowering of E-cadherin is frequently associated with higher tumor grade and poor prognosis in various cancers (21). Given the role of Wnt/β-catenin signaling in CSC maintenance and the miRNAs in regulating wnt pathway, understanding the stepwise regulation of its mediators is crucial (22).

Our recent study has characterized CD44^high^CD24^low^ cells as the potential CSC population in OSCC (2). CD24, a small cell surface protein, was identified as a critical determinant of differentiation in hematopoietic cells and mammary epithelial cells (23). Besides its role in adhesion, cadherin switching and migration, CD24 is involved in diverse signaling networks that promotes oncogenesis or regression (24). Although role of CD44 is well established (3), the involvement of CD24 in determining stemness is less explored, particularly in oral CSCs. In this study, we show that miR-146a confers CD44^high^CD24^low^status to OSCC cells by targeting CD24 (25). We also observed that CD24 downregulation caused by miR-146a leads to β-catenin stabilization through the AKT pathway. We propose that miR-146a/CD24/AKT/β-catenin axis influences the stemness characteristics of oral cancer cells.

## Materials and Methods

### Cell Culture and Transfection

Human OSCC cell lines SCC131, SCC084 and SCC036 were obtained from Dr. Sussane Gollin, University of Pittsburgh (26). SCC131 and SCC036 were derived from new primary tumors while SCC084 from recurrent ones. The characteristics and phenotype of SCC cell lines are described in **Supplementary File 2**, **Table 1**. These cells were maintained in 5% CO_2_ at 37°C in DMEM medium supplemented with 10% fetal bovine serum (FBS) and antibiotics (Life Technologies, Thermo Fisher Scientific Inc., MA, USA). The ATCC (American Type Culture Collection) oral cancer cell line, SCC25 was cultured in complete DMEM-F12 medium and 400ng/ml Hydrocortisone (Sigma Aldrich) under similar conditions. Magnetic assisted cell sorting was used to isolate CD44^high^CD24^low^ cells in a sequential separation method. Briefly, 10^6^ OSCC cells were first incubated with CD24 antibody which got depleted using magnetic bead-based enrichment. The CD24^low^ cells were then incubated with CD44 antibody to enrich CD44 expressing cells i.e. CD44^high^CD24^low^ population. The CD24^high^ population from the previous step was used to obtain the CD44^high^CD24^high^ cells by same method. All these populations were further confirmed and characterized by flow cytometry analysis as shown in our previous work (2). For transfection, Lipofectamine™ 2000 (Invitrogen) was used in serum free medium. Transfected cells were harvested after 48 hrs or 72 hrs for over-expression or knockdown studies respectively.

### Plasmid *C*onstructs, miRNA *i*nhibitors and siRNAs

We obtained mir-146a and mir-146a SDM (mutated by site-directed mutagenesis) expressing pU61 construct from Dr. Nitai. P. Bhattacharjee (SINP, Kolkata). The TOP-flash/FOP-flash reporters, dnTCF4, Numb, and pcDNA3.1 empty vector along with miR-146a promoter LucA, LucB and mLucA Luciferase constructs were kind gifts from Muh-Hwa Yang (Taiwan). Human CTNNB1 expression plasmid deposited by Eric Fearon was purchased from Addgene (#16828). CD24 cDNA cloned into the pCDNA3.1 vector and the full-length 3’-UTR of CD24 cloned into pMIR (Ambion) were obtained from Heike Allgayer (University of Heidelberg, Germany). Anti-miR-146a (ID: AM17120) was obtained from Ambion, CD24 siRNA (a pool of 3 target specific siRNAs), UBE2C siRNA (sc-61742) and scramble siRNA from Santa Cruz. CTNNB1 shRNA constructs (Addgene # 18803) were provided by Dr. Mrinal Kanti Ghosh, IICB, Kolkata. The generation of CTNNB1 shRNA lines are discussed in supplementary methods (**Supplementary File 5**). MiR-146a over-expression cassette was sub-cloned from pU61 into the pLKO.1 TRC vector (Addgene plasmid #10878). Packaging plasmids psPAX2 (Addgene plasmid # 12260) and pMD2.G (Didier Trono, Addgene plasmid# 12259) was used to generate the miR-146a over-expression lentiviral particles and target cells were infected following the manufacturer’s protocol. Stable transduced cells were selected by puromycin (Gibco) and over-expression efficiencies were verified by qRT-PCR and western blotting. CD24 was co-transfected, and clones were selected by G418.

### Quantitative Real Time PCR

TRIzol (Invitrogen, Thermo Fisher Scientific Inc., MA USA) method was used for isolation of total RNA as per manufacturer’s instructions. 250 ng of RNA were converted to cDNAs using stem-loop primers specific for reverse transcription of individual miRNAs (27). MiRNA cDNAs were amplified with forward primers specific for individual miRNAs and a URP (universal reverse primer), with U6 snRNA as an endogenous reference control. For mRNA expression changes, protocol was similar to that described previously (2). SYBR Green master mix (Roche, USA) was used to perform qRT-PCR in the 7500 Fast Real-Time PCR instrument (Applied Biosystems, USA). Relative quantification (2^^–ΔCT^*100) was plotted for most of the q-PCR experiments. In some cases, fold changes (2^–ΔΔCT^) or Log transformed [Log10 (2^^–ΔCT^)] values were calculated and plotted. The delCts or log fold changes were subjected to unpaired t-tests in GraphPad prism 8. All analysis was done using results from three independent experiments taking mean values of at least 2 technical replicates from each experiment. Technical replicates include pipetting repetitions for q-PCR. Primer sequences of genes, miRNA forward and loop primers are listed in **Supplementary File 1**.

### Analysis of TCGA and NCI-60 Datasets

RNA and miRNA-seq data were acquired for a total of 292 HNSCC tumor specimens from TCGA (The Cancer Genome Atlas) data portal (https://tcga-data.nci.nih.gov/tcga/). The percentage of CSCs can vary largely depending on the origin, stage, location, age and a number of other physiological attributes associated with a tumor. Since the TCGA data is obtained from bulk tumor, average expression values of CD44 and CD24 across these samples were found to be extremely heterogenous and so was miR-146a levels. To eliminate the discrepancy of medium expression levels and compare between relatively pure CSC and non-CSC populations, we first grouped top 25% of CD44 high and CD44 low expressing tumors and then further sub-grouped 25% of these tumors based on CD24 expression. These samples (n=19) were designated as CD44^high^CD24^low^ and CD44^low^CD24^high^, wherein we checked the differential expression of miR-146a and calculated statistical significance using R Limma Package (unpaired t-test). Node status of these patients was also correlated with miR-146a expression using GraphPad Prism5 software. NCI-60 miRNA expression dataset (GEO accession number GSE26375) was analyzed to compare the miR-146a expression between the epithelial and mesenchymal groups as classified earlier (16) using Mann Whitney’s u test.

### Flow Cytometry

CD44-PE and CD24-FITC (BD Pharmingen) conjugated antibodies were used for double staining of miR-146a transfected cells. Cells were then washed and subjected to flow cytometry on the BD LSRFortessa and analyzed using BD FACSDiva 6.2 software. Isotype controls were included for the non-specific staining. The gating strategy for the flow analysis has been schematically described in **Figure S2A**. Relative fluorescence intensity of CD44 and CD24 for different experimental conditions were plotted respectively and p-values were calculated by one-sample t-test on log fold change in GraphPad prism 8.

### Sphere Forming Assay

MiR-146a transfected cells were trypsinised and a single cell suspension was ensured. Low attachment 6-well plate were used for re-seeding the cells at a density of 5000 cells/ml in DMEM-F-12 serum free media containing 1% B27 supplement, 20 ng/ml of EGF and 20 ng/ml of bFGF (Invitrogen). 500 µl of media was added every 2-3 days. Photographs of the spheres were taken under inverted microscope (Leica TCS SP8; Germany) with 20X magnification at 7-14 days. All experiments were done in biological triplicates. Spheres were counted for a number of fields shown as overlaid dots onto the bar graphs and statistical significance was determined by Mann-Whitney’s unpaired-test in GraphPad prism 8.

### Immuno-Fluorescence

Sorted populations of SCC131 were grown on cover-slips overnight and then fixed with chilled aceto-methanol (1:1). 0.03% Saponin (Calbiochem, Germany) was used for permeabilization followed by blocking with 3% BSA. Rabbit monoclonal antibody against β-catenin and mouse monoclonal antibody against CD24, CD44 (Cell signaling technology) were added at a dilution of 1:200 and incubated overnight. It was then probed with anti-rabbit-FITC and anti-mouse Alexa-Flour 633nm conjugated secondary antibody (molecular probes) and counter stained with DAPI (Invitrogen) for nuclear staining. Images were taken under a confocal microscope (Andor Spinning Disc Confocal Microscope, Andor Technology, Belfast, Ireland) at 60X magnification.

### Western Blotting

Cell lysates were prepared after 48 hrs of transfection in NP-40 lysis buffer (Invitrogen) and protease inhibitor cocktail (1X). Equivalent amounts of denatured protein samples were subjected to SDS-PAGE (8%-10%), separated by size and transferred on to PVDF membrane (Millipore, Billerica, USA). Antibodies used for immuno-blotting were polyclonal β-catenin, E-cadherin, CD44 and CD24, Involucrin (Santa Cruz Biotechnology, CA, USA), polyclonal Oct4 and Sox2 (Abcam), polyclonal C-myc, Akt and phospho-Akt (Cell Signaling Technology, USA) and UBE2C (Abcam). Bands were obtained using ECL substrate (Thermo Scientific, USA) from HRP-conjugated secondary antibody (Sigma). Proteasome Inhibitor MG132 (Calbiochem) and Akt inhibitor LY294002 (Cell signaling Technology, USA) were both used at a concentration of 50 µM. Transfected cells were treated for 4 hours before harvesting. Band intensities of each protein were analyzed by ImageJ to obtain densitometric values for their quantification. These were normalized to β-actin for individual experimental sets and fold change calculated. All the histograms were expressed as means ± S.D. of three different experiments and p values computed in GraphPad Prism 5 (Student’s two tailed t test).

### *In Vivo* Tumor Xenograft Experiments

Animal experiments were performed following guidelines of the Institutional Animal Ethics Committee (IAEC) of National Centre for Cell Science, Pune. All the animals were issued under the project IAEC/2012/B183. To investigate the effect of miR-146a overexpression on OSCC growth *in vivo*, 3×10^6^ empty vector- and microRNA overexpression construct-containing SCC084 cells were injected subcutaneously into the dorsal flanks of eight NOD/SCID male mice (18 weeks old) on left and right side respectively. When palpable tumors could be seen, the mice were segregated into groups of four each. Mice in one of the groups were injected with 25 mg/kg of body weight of Quercetin (Sigma) on every alternate day for a period of 15 days. The experiment was terminated when the average miR-146a over-expressing SCC084 tumor volumes in the group which received no quercetin reached about 1200 mm^3^. At the termination of the experiment, the animals were sacrificed by CO_2_ asphyxiation and the tumors were collected for further analysis. Tumor diameters were measured each time the quercetin was injected and at the termination of the experiment using digital Vernier Caliper. Excised tumor tissues were weighed and then stored in RNAlater solution (ThermoFisher Scientific) in -20°C freezer. Tumor volumes were determined using the following formula: π/6[(d1×d2)^3/2^]; where d1 and d2 are two different diameters of a tumor. In another experiment, to investigate effect of simultaneous overexpression of miR-146a and CD24 on OSCC growth *in vivo*, 3×10^6^ empty vector- and miR-146a and CD24 overexpression constructs-containing SCC084 cells were injected subcutaneously into the dorsal flanks of four NOD/SCID male mice (15 weeks old) on left and right side respectively. Tumor volumes were measured when palpable growth could be observed. The experiment was terminated when tumor volumes reached 1300 mm^3^. The animals were euthanized by CO_2_ asphyxiation and the tumors were collected. Tumor tissues were processed as described previously for the other experiment. Tumor volumes were calculated intermittently before final termination and a 2-way ANOVA was performed to determine p-value that consider difference at all timepoints in GraphPad prism 8. Tumor weights were measured at end point and unpaired student’s t-test was done to calculate significance.

### Immunohistochemistry

Formalin fixed xenograft tumors were embedded in paraffin and 5µm sections were cut for immunohistochemical staining. The sections were baked (stretched) at 65°C for 20 minutes, deparaffinized in xylene and rehydrated in grades of ethanol. Heat mediated antigen retrieval was performed in citrate buffer (10mM sodium citrate, 0.05% Tween 20, pH 6.0) followed by quenching of the endogenous peroxidase activity by 0.3% H_2_O_2_ for 7 minutes. Beta-catenin antibody (sc-7199) was used at 1:100 dilution and incubated overnight at 4°C. Next day, the primary antibody was washed, and the sections were probed with HRP-conjugated secondary antibody (Sigma). The antigen-antibody complexes were visualized using diaminobenzidine (D8001, Sigma Aldrich). The sections were counterstained with Mayer’s hematoxylin, dehydrated in ethanol series and mounted using DPX. Images were acquired using Lawrence and Mayo’s LM-52-1704 binocular microscope using 40X objective and processed using the ScopeImage 9.0 software.

### Reporter Assays

Cells seeded in 24 well plates were co-transfected with miR-146a OE plasmid and either CD24 3’UTR or miR-146a promoter luciferase construct using Lipofectamine™ 2000 (Invitrogen). The TOP-Flash and FOP-Flash reporters were also used under similar conditions. Promega dual luciferase assay system was performed according to the manufacturer’s protocol. After 48 hr of transfection, medium was washed off with 1x PBS and cells were lysed with Passive Lysis Buffer (Promega) and luminescence was measured in Promega Glomax 20/20 luminometer. The luminescence values were transfection normalized with the internal control pRL-TK (50 ng, Renilla Luciferase; Promega). Experiments were performed with three biological replicates. One-sample t-test (on log fold change of control vs experiment) were performed to calculate p-value in GraphPad prism 8.

### Chromatin Immunoprecipitation

Cells seeded in 10cm dishes were transfected with either Scr or CD24 siRNA for endogenous ChIP on the miR-146a promoter. For transient ChIP assays, miR-146a LucA/m-LucA/LucB promoter with or without CD24 over-expression was used. After 48hrs, 1X formaldehyde solution was added for DNA-protein crosslinking. Cells were lysed in SDS lysis buffer followed by sonication in Bioruptor (Diagenode) to obtain 200-1000 bp chromatin fragments. ChIP dilution buffer was used to dilute the sheared chromatin followed by preclearing with Protein G Agarose beads (Sigma) for 30min. After preclearing, 20% of the lysate was kept aside as the input and the remaining was divided equally for IP and IgG. Immunoprecipitation was carried out using 5µg of β-catenin (Santa Cruz) and normal IgG control (Sigma) and incubated overnight. The following day, Protein G Agarose beads were added to collect the Antibody/Antigen/Chromatin complex. The complex was washed briefly with cold low salt immune complex wash followed by high salt immune complex buffer, lithium chloride immune complex buffer and Tris-EDTA buffer. It was then reverse-crosslinked and the DNA purified using Phenol/Chloroform extraction method. PCR amplification of the immunoprecipitated DNA was carried out using primers listed in **Supplementary File 1**. Composition of the ChIP buffers are provided in **Supplementary Methods** (**Supplementary File 5**).

### Statistical Analyses

Two to three experimental repetitions consisting of at least three biological replicates were used in the study for statistical inference. Various experimental data were subjected to an independent two-tailed Student’s *t* test; one-sample-t-test or unpaired t-tests (with either equal or unequal variance) to measure the significance value as was applicable. We confirmed that data must be normally distributed for p-value calculations by t-test or ANOVA, In case of fold changes, we have log transformed the data, beforehand to approximate normal distribution. One-sample t-tests have been used instead of usual two-sample t-tests in case of ratio data, using log FC (fold change) of control vs experimental group. Non-parametric tests were used for the data that are not normally distributed.

All the statistical tests were performed using graph-pad prism and their versions are mentioned in the respective methods as well as legends. In some cases, R package version 3.5.0 was used to generate the correlation graphs and calculate p values. For miRNA discovery, we used multiple correction analysis as shown in **Supplementary File 2**, **Table 2**, and the selection was based on FDR cut off for adjusted p <0.05. Individual data points used are shown onto the respective bar graphs and p-values indicated.

## Results

### MiR-146a Is Over-Expressed in CD44^high^CD24^low^ Population of OSCC Cell Lines and Primary Tumors

**Figure 1:**
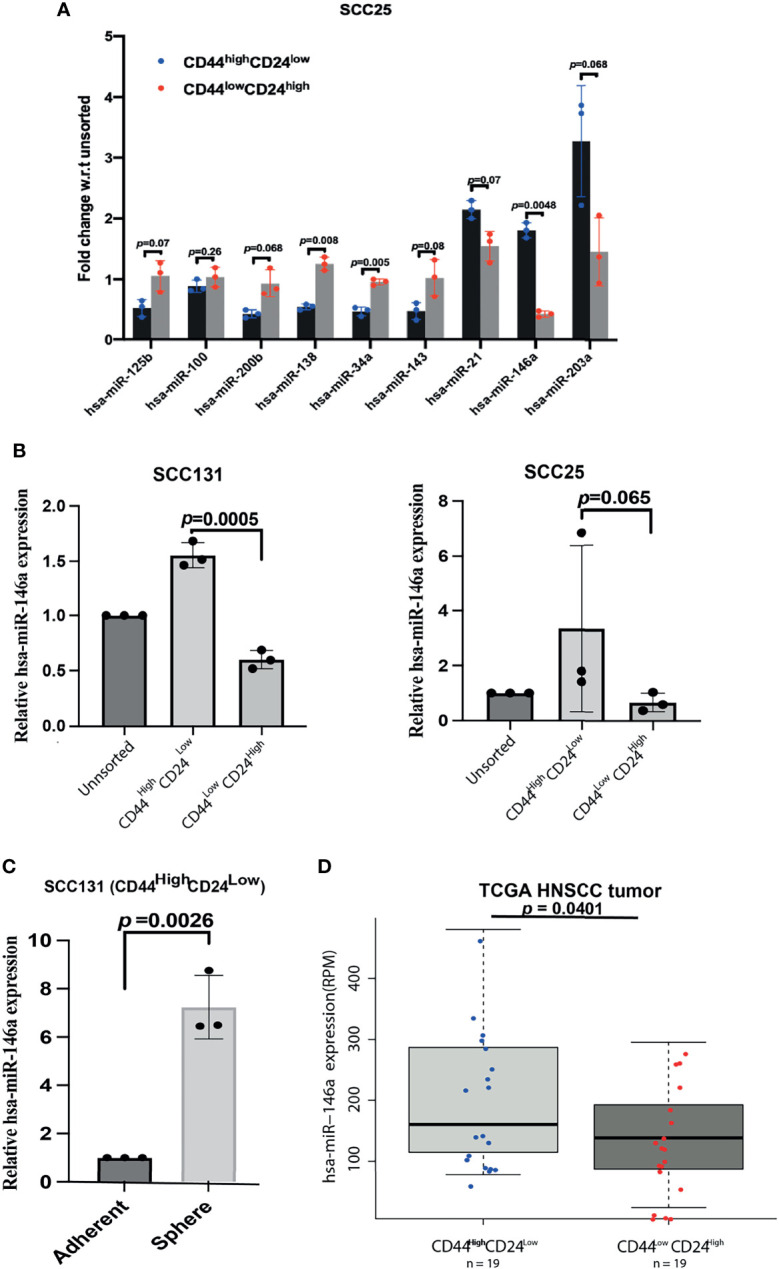
Over-expression of miR-146a in CD44^high^CD24^low^ cells of Oral Squamous Cell Carcinoma. **(A)** Total RNA extracted from the stem (CD44^high^CD24^low^) and the non-stem (CD44^low^CD24^high^) sub-populations of SCC25 cells were reverse-transcribed using stem-loop primers specific for subsequent Real time PCR analysis of various miRNAs using respective forward primers and Universal reverse primer (**Supplementary File 1**). Fold changes w.r.t the unsorted population have been plotted and their adjusted p-values are shown on respective miRNAs (calculated using multiple correction FDR analysis as shown in **Supplementary File 2**, **Table 2**). **(B)** Expression of miR-146a was re-analyzed in UPCI: SCC131 and UPCI: SCC25 by qRT-PCR. Fold change values were plotted and log FC subjected to unpaired t-tests to calculate *p*-values using GraphPad prism 8. **(C)** Quantification of miR-146a transcripts in the spheres enriched from CD44^high^CD24^low^ cells of SCC131 compared to that grown under differentiating adherent conditions. Data is representative of 3 independent experiments and fold change is shown with mean ± SD (p-value calculated on log FC). U6snRNA was used to normalize relative expression values. **(D)** Box-Scatter plot showing the differential expression of miR-146a in the CD44^high^CD24^low^and CD44^low^CD24^high^ subgroup of HNSCC tumors obtained from TCGA. (p-value from unpaired t-test was calculated in R package to show statistical significance).

To identify the cellular miRNAs regulating CSC phenotype of OSCC cells, we initially screened nine miRNAs that are aberrantly expressed in human cancers with their reported role in cancer stemness and EMT (14–18). The expression of these miRNAs was investigated in the CD44^high^CD24^low^ (CSCs) and CD44^low^CD24^high^ (non-CSCs) populations of SCC25 cells, which were purified and characterized as previously described (2). QRT-PCR data showed significant difference in the expression of, miR-138, miR-34a, and miR-146a between CD44^high^CD24^low^ and CD44^low^CD24^high^ population of SCC25 cells (**Figure 1A**). Amongst them, we focused on miR-146a in view of its context dependent role in various cancers (19, 28, 29). MiR-146a is consistently over-expressed in oral CSCs (18, 19), therefore it was intriguing to explore its possible connection with stemness and the underlying mechanisms. Up-regulation of miR-146a was further confirmed in the CD44^high^CD24^low^ population of SCC131 and SCC25 cell lines (**Figure 1B**). MiR-146a expression was found to be increased in the sphere forming culture conditions of SCC131 derived CD44^high^CD24^low^ cells, suggesting it as an important determinant of oral cancer stemness and maintenance (**Figure 1C**). Increased miR-146a expression has been earlier shown to predict poor survival of OSCC patients (18). Interestingly, the analyses in TCGA Head and Neck Squamous Cell Carcinoma (HNSCC) patient’s cohort (30), showed increased miR-146a expression in patients with CD44^high^CD24^low^profile compared to those with CD44^low^CD24^high^, complementing our cell line data (**Figure 1D**). While there was not much difference in the histological stage of the tumors across the two categories, most of the CD44^low^CD24^high^ tumors were free of lymph node metastasis (**Figure S1A**, **Supplementary File 3**). Moreover, miR-146a expression of the node positive patients was relatively higher than that of the node negative ones, although not statistically significant (**Figure S1B**). Together, our data suggests possible correlation of high miR-146a expression with CSC-like phenotype in oral tumors.

### Ectopic Expression of miR-146a Induces CSC Characteristics

**Figure 2:**
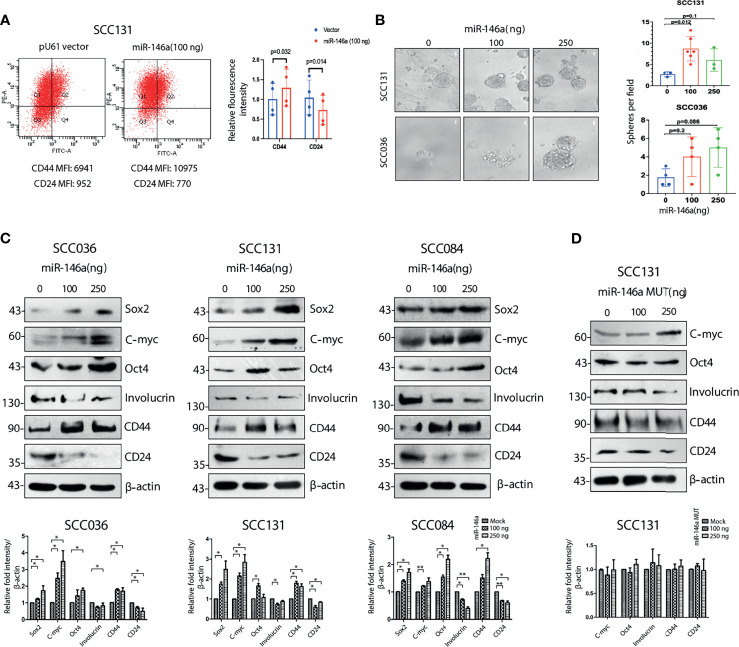
Cancer stem cell characteristics induced by miR-146a in OSCC cell lines. **(A)** MiR-146a transfected SCC131 cells were analyzed by flow cytometry and fold change for the respective mean fluorescence values of CD44 (PE) and CD24 (FITC) have been plotted (p values calculated using one sample t-test on logFC). **(B)** Equal number of vector and miR-146a transfected UPCI: SCC131 and UPCI: SCC036 cells were seeded in ultralow-attachment 6-well plate at clonal density. Sphere forming structures was captured at five random fields at 20X magnification using phase contrast microscope (Leica CTR4000) with scale bar equal to 50 µm. Number of spheres per field were counted and plotted (p-value calculated by Mann whitney’s u test in GraphPad prism 8). **(C)** Representative images of western blots showing dose dependent increment of Sox2, C-myc, Oct-4, Involucrin and CD44, decrease in CD24 in the UPCI: SCC036, UPCI: SCC131 and UPCI: SCC084 upon ectopic expression of miR-146a. β-actin bands was used to normalize the data. Band intensities of each protein has been quantified from three biological replicates and the average ± sd is plotted in GraphPad prism 5 showing statistical significance in the respective graphs (below) (*P < 0.05, **P < 0.01) **(D)** Similar western blots upon transfection of miR-146a with mutated seed sequences in UPCI : SCC131 and its graphical representation (below).

We next investigated whether ectopic expression of miR-146a affect the proportion of CSCs by flow cytometry and found a significant increase in the relative proportion of CD44^high^CD24^low^ population in SCC131 cells (**Figure 2A**). Similar results were also obtained with SCC036, SCC084 and SCC25 cells, respectively (**Figure S2B**). Characteristic sphere forming ability of miR-146a expressing SCC131 and SCC036 cells were also markedly enhanced (**Figure 2B**). In several studies, the cancer stem cells have been found to express Yamanaka factors which are indicators of stemness, and show loss of epithelial differentiation markers (31). We also observed that ectopic expression of miR-146a led to the increased expression of intracellular stem cell markers such as Oct4, Sox2 and C-myc and loss of Involucrin (32) (**Figure 2C**). However, in SCC131 cells the levels of Oct4 and Involucrin did not show a dose dependent change upon miR-146a over-expression (**Figure 2C**). Additionally, a pronounced decrease in CD24 protein levels upon ectopic miR-146a expression was evident in SCC036, SCC131 and SCC084 cells (**Figure 2A**, **S2B** and **2C**). Altered expression of CSC markers upon knockdown of miR-146a was also evident in SCC131 cells (**Figure S2C**). The modulation in the expression of miR-146a upon ectopic expression or knockdown was validated by qRT-PCR (**Figures S2D, E**). Transfection of miR-146a containing mutated seed sequence, however, did not alter the levels of stem-related proteins in a statistically significant manner (**Figure 2D**). These results demonstrate that miR-146a contributes to enrichment of CSCs in OSCC through increased expression of stem cell markers and lowered CD24 levels.

### MiR-146a Activates Wnt/β-Catenin Pathway and Promotes EMT in Oral CSCs

**Figure 3:**
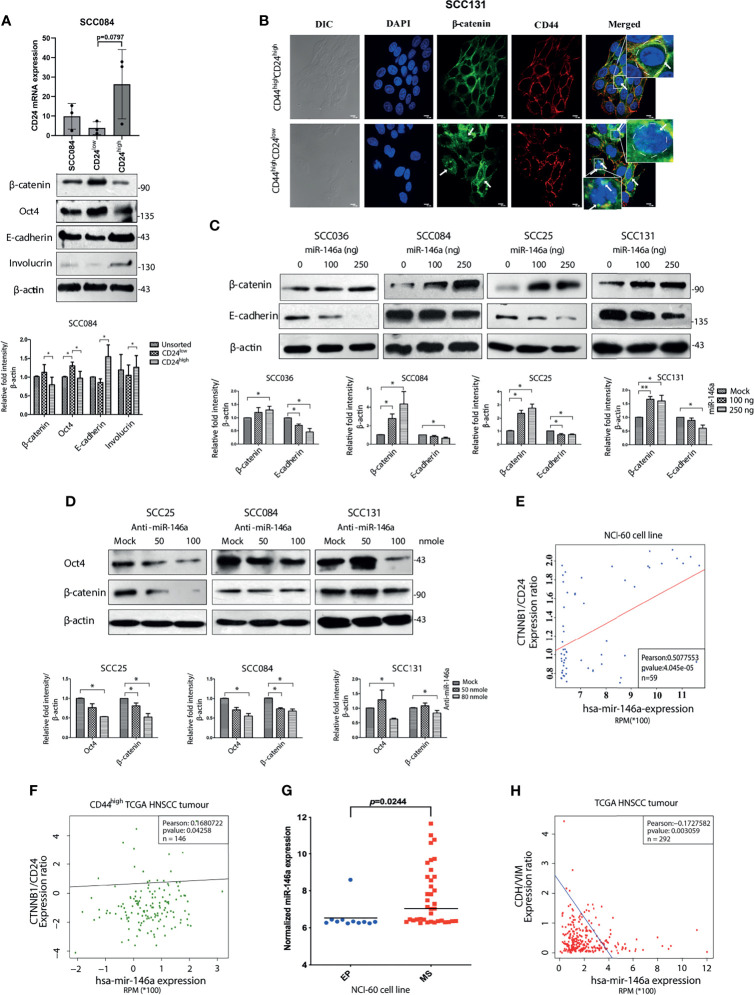
MiR-146a induced β-catenin/Wnt Signaling in CD44^high^CD24^low^ population. **(A)** Validation of CD24^low^ and CD24^high^ cells from SCC084, as shown by qRT-PCR of CD24 (unpaired t-test on delCT values). 18srRNA served as an endogenous control. Western blot images of β-catenin, Oct-4, E-cadherin and Involucrin in the respective populations along with its quantitative plot indicating the statistical data. **(B)** Representative confocal immunofluorescence images (60X magnification) of CD44^high^CD24^high^ and CD44^high^CD24^low^ subpopulation of SCC131 showing β-catenin (green) and CD44 (red) counterstained with DAPI (blue) (scale bar equal to 10 µm). Enlarged image is shown in inset. **(C)** Each of the SCC cell lines were subjected to western blot analysis of β-catenin and E-cadherin upon increasing doses of miR-146a. **(D)** β-catenin and Oct-4 immunoblotting upon increasing doses of anti-miR-146a. Data normalized with β-actin. All the data has been graphically represented beneath the respective figures (*P < 0.05, **P < 0.01, ***P < 0.001). **(E)** Association of miR-146a expression with β-catenin/CD24 in the NCI-60 cell lines (n = 59) and **(F)** with β-catenin/CD24 ratio in the CD44^high^ tumors of the TCGA HNSCC patients (n = 146). Statistical significance was determined by Pearson correlation test. Pearson correlation coefficient is shown in each plot. **(G)** Box plots showing miR-146a expression in NCI-60 cell lines classified as epithelial (EP) and mesenchymal (MS) subgroups. p value has been calculated using mann-whitney’s u-test using GraphPad prism 8. **(H)** Correlation (Pearson) of miR-146a with CDH1/VIM expression ratio based on the RNA-Seq data from 292 TCGA HNSCC specimens.

It is known that Wnt, Notch and Hedgehog signaling pathways are often involved in self-renewal property of stem cells and hence its niche maintenance (33). Accordingly, we observed increased expression of β-catenin and Cleaved Notch1 in CD44^high^CD24^low^ population of SCC25 and SCC131 cells (**Figure S3A**). We also observed higher expression of β-catenin at RNA level in CD44^high^CD24^low^ cells compared to CD44^low^CD24^high^ cells (**Figure S3B**). To specifically decipher the role of CD24, we began to compare the CD44^high^CD24^low^ population with the CD44^high^CD24^high^ cells only (34). Interestingly, β-catenin protein levels were remarkably high in SCC084 CD24^low^ compared to CD24^high^ cells that correlated well with the expression of stemness markers in these cells (**Figure 3A**). We have previously reported the purity levels of these sub-populations and shown that CD44^high^CD24^high^ cells are less stem-like than the CD44^high^CD24^low^ ones (2). In addition, we detected nuclear localization of β-catenin in the CD44^high^CD24^low^ population of SCC131 cells, whereas it remained membrane bound in CD44^high^CD24^high^cells (**Figure 3B**). Enhanced transcriptional activity of β-catenin in CD44^high^CD24^low^ cells compared to CD44^high^CD24^high^ was observed by measuring the relative wnt reporter activity in the respective cell populations of SCC084 (**Figure S3C**). Interestingly, upon stable knockdown of β-catenin, not only the stem cell markers were reduced but also a modest increase in CD24 expression was observed along with E-cadherin (**Figure S3D**). These results suggest a possible cross talk between CD24 and β-catenin in conferring stemness and EMT to these cells.

The clue that β-catenin level might influence stemness in OSCC cells led us to investigate whether miR-146a directly regulates β-catenin. We did find that over-expression of miR-146a lead to the dose dependent increment in β-catenin levels with concordant decrease in E-cadherin (**Figure 3C**). It is already known that miR-146a targets the 3’UTR of Numb, a protein that promotes lysosomal degradation of β-catenin and Cleaved Notch1 (35). We indeed, confirmed reduced levels of Numb upon miR-146a over-expression along with stabilization of Cleaved Notch1 (**Figure S3E**). Conversely, inhibition of miR-146a activity led to β-catenin degradation along with the loss of Oct4 as expected of its ability to alter stem cell markers (**Figure 3D**). Notably, we did not observe these changes upon mutant miR-146a over-expression indicating that this effect of miR-146a is sequence specific (**Figure S3F**). To emphasize the contribution of β-catenin in miR-146a induced stemness, we transfected miR-146a in β-catenin shRNA expressing cells and found no change in expression of CSC markers (**Figure S3G**) as compared to that of non-silencing controls. Strikingly, the ability of anchorage independent growth of SCC131 cells induced by miR-146a was also reduced in the absence of β-catenin (**Figure S3H**) suggesting that the tumorigenic role of miR-146a in OSCC is β-catenin dependent.

To show the generality in the relationships among miR-146a, CD24 and β-catenin, we checked the correlation between miR-146a and β-catenin/CD24 expression across the NCI-60 cell lines and found it to be positively correlated (**Figure 3E**). Similarly, examination of CD44^high^ HNSCC tumors from TCGA dataset revealed a positive correlation between miR-146a expression and β-catenin/CD24 ratio, showing their clinical relationship (**Figure 3F**). Further, the observed down-regulation of E-cadherin upon miR-146a expression prompted us to address the miR-146a driven EMT phenomenon in OSCC. Indeed, miR-146a was found to be significantly over-expressed in the mesenchymal (MS) cell lines showing higher CD44 and lower CD24 expression (2), compared to the epithelial (EP) cell lines of the NCI-60 panel (**Figure 3G**) (16). In addition, the miR-146a expression in TCGA tumor samples was negatively correlated with the E-cadherin to Vimentin ratio (**Figure 3H**). Based on these observations, we propose that miR-146a induced stemness and EMT in OSCC is potentially mediated through lowering of CD24 followed by activation of β-catenin.

### MiR-146a Targets CD24 in Oral CSCs

**Figure 4:**
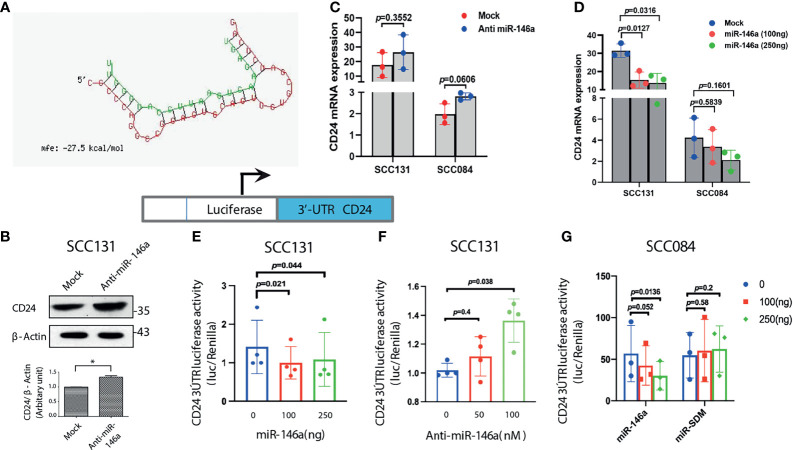
MiR-146a targets CD24 post-transcriptionally. **(A)** Secondary structure prediction of CD24 3’UTR base pairing with the seed sequences of miR-146a with negative free energy allowing favorable binding. **(B)** Immunoblotting of CD24 in SCC131 cells subjected to miR-146a knockdown showing significant up-regulation upon normalization with β-actin. **(C)** qRT-PCR to detect CD24 transcripts in the anti-miR-146a treated or **(D)** miR-146a over-expressed SCC131 and SCC084 cells.18srRNA served as an endogenous control and p-values calculated by unpaired t-test (n = 3) on delCT values that was plotted. Schematic representation of the CD24 3’UTR luciferase constructs (below). **(E)** CD24 3’UTR reporter activity upon miR-146a over-expression and **(F)** miR-146a knockdown in SCC131 cells. **(G)** Luciferase activity of the CD24 3’UTR reporter gene in SCC084 cells with increasing expression of miR-146a or miR-SDM (site-directed mutant miR-146a). Data in **(E–G)** are mean ± SD, normalized with pRL-TK vector and statistical significance was measured by one sample t-test in GraphPad prism 8, from three or four independent experiments.

Since, we observed a negative correlation between miR-146a and CD24 expression both experimentally and in patient cohort analysis, we assumed CD24 as a putative target of miR-146a through which it might impart stemness in OSCC. Although, *in silico* identification of miRNA targets using the prediction software did not reveal CD24 as the probable target, we did find matching of miR-146a seed sequence in the CD24 3’UTR in miRanda (**Supplementary File 4**). Despite one mismatch, the maximum free energy of miRNA-mRNA binding was favorable enough for hybridization and targeting (**Figure 4A**). In **Figure 2C**, we had already examined that CD24 expression was significantly depleted upon miR-146a transfection in a dose dependent manner in SCC036, SCC131 and SCC084 cells. Alongside, it was also up-regulated upon inhibition of miR-146a in SCC131 cells (**Figure 4B**). We also observed an expected trend in CD24 transcript levels in both SCC131 and SCC084 upon modulation of miR-146a, although it was not statistically significant in some cases (**Figures 4C, D**). To further confirm that CD24 is a direct target of miR-146a, we co-transfected luciferase reporter vector containing the 3’UTR fragment of CD24 gene with either miR-146a expressing vector or anti-miR-146a in SCC131 cells. As shown in **Figures 4E, F**, miR-146a over-expression reduced the luciferase activity of CD24 3’UTR, while miR-146a inhibitor elevated the same. On the contrary, transfection with mutated mir-146a did not alter the CD24 3’UTR luciferase activity significantly (**Figure 4G**). Thus, this data experimentally validates the ability of miR-146a to directly target CD24 gene by binding to its 3’UTR. This justifies the involvement of miR-146a in negative regulation of CD24 expression in oral cancer stem cells.

### MiR-146a Stabilizes β-Catenin by Down-Regulating CD24

**Figure 5:**
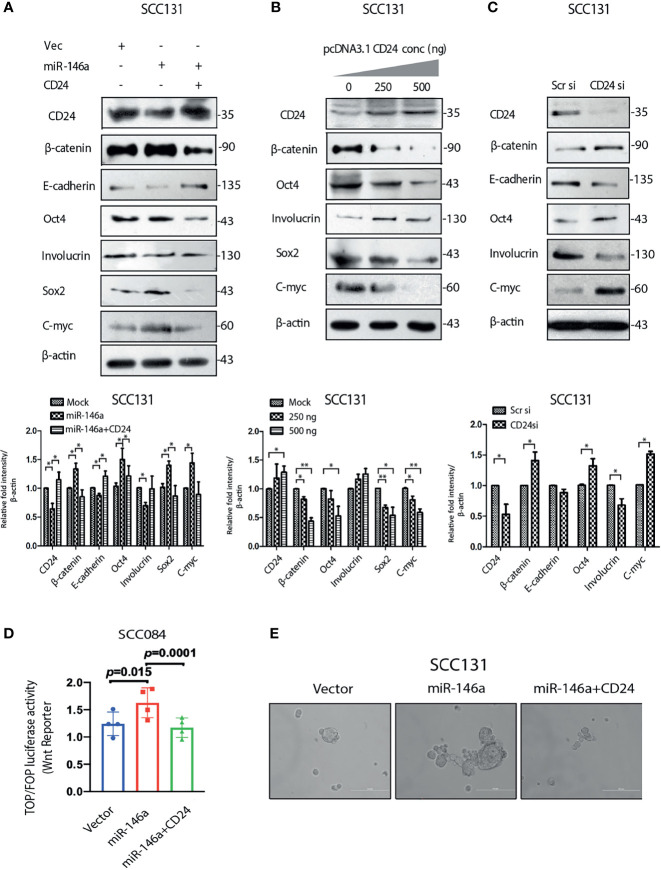
MiR-146a promotes stemness by down-regulating CD24 **(A)** Immunoblot analysis of SCC131 transfected with either a vector control, miR-146a with or without CD24 showing rescued expression of β-catenin and E-cadherin as well as Involucrin Oct-4, Sox2, C-myc. **(B)** Immunoblotting of the same proteins with increasing dose of CD24 expression and **(C)** upon knockdown of CD24 in SCC131 cells. β-actin is loaded as an endogenous control. Histograms show fold changes in the densitometric values of band intensity and shown as means ± S.D. of 3 individual experiments. **(D)** Wnt reporter activity as measured by Top-Flash vs Fop-Flash luciferase construct in SCC084 mock, miR-146a with or without CD24 transfection, as indicated (p-value computed by one sample t-test). **(E)** Sphere formation ability under similar conditions as described in **(D)** (Scale bar = 200 µm).

We documented in the previous sections that miR-146a expression enhances β-catenin level (**Figure 3**) and miR-146a targets CD24 (**Figure 4**). To gain mechanistic insight into the miR-146a mediated β-catenin stabilization, CD24 was over-expressed in miR-146a over-expressing cells. Interestingly, CD24 not only abolished the stemness markers but also the expression of β-catenin and CD44 (**Figure 5A** and **Figure S4A**). This indicated that down-regulation of CD24 by miR-146a (**Figure 4**) was instrumental in maintaining high β-catenin levels (**Figure 3**) and consequently the downstream stemness phenotype. This was further exemplified as CD24 over-expression alone could lead to decreased expression of β-catenin protein and the associated stem cell markers, while the siRNA mediated knockdown of CD24 showed an opposite effect (**Figures 5B, C**). Wnt target gene *C-myc* was significantly depleted upon CD24 over-expression in cells expressing miR-146a, although β-catenin mRNA levels remain unchanged (**Figure S4B**). Other wnt targets, *CD44* and *CCND1* were also altered however were not significant at the level of p value<0.05. (**Figure S4B**). These observations suggest an inverse correlation between CD24 and β-catenin signaling in OSCC cells. It was further confirmed by the inhibition of miR-146a-induced β-catenin nuclear mobilization upon ectopic expression of CD24 (**Figure S4C**). In addition, miR-146a driven increased wnt reporter activity was found to be reduced upon CD24 over-expression (**Figure 5D**). To further investigate its downstream effect on stemness phenotype, we performed an *in vitro* sphere-formation assay. We observed a considerably defective spheroid forming ability of miR-146a transfected cells in the presence of CD24 (**Figure 5E**). Together, these observations suggest the possible contribution of CD24 in regulating Wnt pathway through β-catenin, thereby affecting CSC-like traits.

### Involvement of pAKT in CD24 Mediated Degradation of β-Catenin

**Figure 6:**
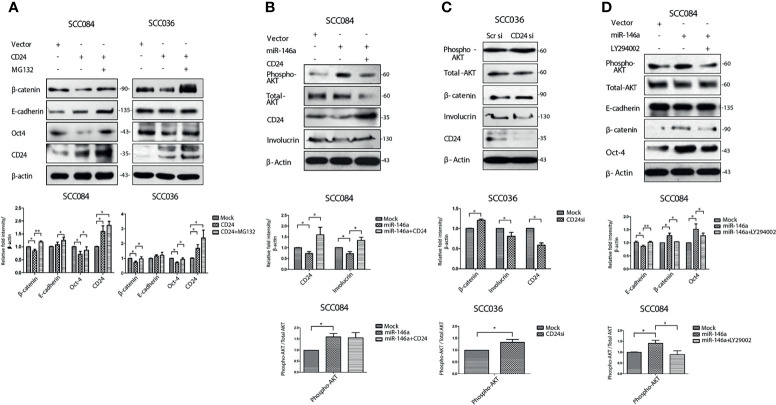
MiR-146a leads to AKT mediated stabilization of β-catenin. **(A)** Western blot analysis revealed degradation of β-catenin upon CD24 over-expression revived with MG132 treatment in SCC084, SCC036. **(B)** Phospho-AKT levels upon ectopic expression of miR-146a alone or in combination with CD24. Total AKT is also shown. **(C)** Phospho-AKT and Total AKT levels upon siRNA mediated down-regulation of CD24 in SCC036 cells. **(D)** Effect of pAKT inhibitor (LY294002) on β-catenin levels in SCC084 cells. Histograms showing fold change in the densitometric values of band intensity is represented as avg ± S.D. of n different experiments (n = 3) (*P < 0.05, **P < 0.01).

Next, to elucidate the cause of β-catenin reduction in the presence of CD24, we treated SCC084 and SCC036 cells with MG132, a proteasomal inhibitor and found β-catenin levels returned to that of un-transfected controls (**Figure 6A**). This confirms that unlike Numb, which is known to degrade β-catenin lysosomally (35), CD24 degrades β-catenin in a proteasomal dependent manner. The restored β-catenin also re-established the expression of stem cell marker Oct4 irrespective of CD24 over-expression in both SCC036 and SCC084 cells (**Figure 6A**), suggesting that CD24 acts upstream of β-catenin stabilization. E-cadherin levels, however, remained high in the presence of CD24, irrespective of β-catenin stability (**Figure 6A**). Notably, CD24 did not affect Numb expression, corroborating the independent participation of CD24 in regulating β-catenin (**Figure S5A**). To gain mechanistic insights into the CD24 mediated β-catenin degradation, we speculated that CD24 may lead to β-catenin destabilization *via* AKT-GSK-3β pathway (36, 37). Towards this, we did find that CD24 over-expression rescued the miR-146a mediated increase in pAKT (Ser 473) levels (**Figure 6B**), although it remained stable during MG132 treatment (**Figure S5B**). It may be noted that over-expression of CD24 alone caused direct down-regulation of total AKT protein resembling mTOR mediated targeting (38) (**Figure S5C**). Hence the pAKT/AKT ratio remains unchanged upon CD24 expression as noted in **Figure 6B**. On the contrary, knockdown of CD24 in SCC036 increased pAKT and β-catenin levels (**Figure 6C**). We confirmed that CD24 siRNA targeting was specific by checking the effect of an unrelated siRNA UBE2C upon the stem cell markers (**Figure S5D**). Moreover, pAKT was found to be accumulated in the CD44^high^CD24^low^ fraction of SCC25 cells thereby indicating its prior involvement in stemness (**Figure S5E**). In addition, we observed significant depletion of β-catenin upon treatment with pAKT inhibitor (LY294002) which again confirmed its regulation by miR-146a-CD24 pAKT axis (**Figure 6D**).

**Figure 7:**
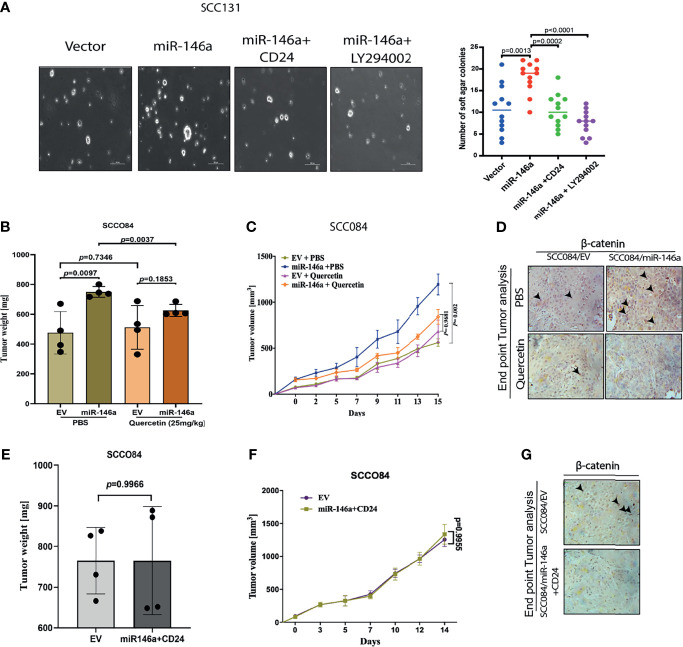
MiR-146a promotes *in vivo* tumor growth which is rescued upon CD24/AKT modulation **(A)** Representative pictures of the colonies from the soft agar assay performed under given transfections and treatment conditions as shown (Left) (Scale bar=100 µm). Number of similar sized colonies were counted for each field and avg ± sd were plotted and graphically represented. *p-*values are measured by mann-whitney u- test between 2 independent experiments (* P < 0.05). **(B)** Bar graphs showing relative weight (mg) of the xenograft tumors generated from control or miR-146a expressing SCC084 treated without or with Quercetin. Data represent mean ± SD (n = 4). P-values were computed using unpaired t-test. **(C)** Line graph showing relative growth rate of tumors in response to Quercetin in SCC084 cells harboring either control vector or stably expressing miR-146a. Once tumors reached a palpable size, one set of mice were injected with Quercetin (10 mg/kg) intraperitoneally and after 10 successive treatment, change in tumor volume was measured at regular interval up to 20 days. Data represent mean ± SD (n = 4) and *p*-values shown using 2-way ANOVA in GraphPad prism 8. **(D)** Immunohistochemistry showing nuclear β-catenin levels in the xenograft tumors obtained from the experiment described in b) (scale bar = 20 μM). **(E)** Bar graphs showing relative weight (mg) of the tumors described in xenograft tumors generated from SCC084 harboring either control vector or stably expressing miR146a and CD24. Data represent mean ± SD (n = 4). P-values were assessed using unpaired t-test **(F)** Line graph showing relative growth rate of tumors described in **(E)**. Data represent mean ± SD (n = 4). For all the time-course experiments, *p*-values were calculated using ANOVA in graph-pad prism8. **(G)** Immunohistochemistry showing nuclear β-catenin levels in the xenograft tumor obtained from the experiment described in **(E)** (scale bar = 20 μM).

This was further supported by the soft agar tumorigenesis assays, which clearly showed that CD24 over-expression or AKT inhibition, both reduced the miR-146a induced colony formation in OSCC cells (**Figure 7A**). These cell line related observations was further validated using mouse xenograft model. To check the effect of miR-146a on *in-*vivo tumor formation, SCC084 cells harboring either an empty vector (SCC084/EV) or stably expressing miR-146a (SCC084/miR-146a), were generated and the over-expression of miR-146a with subsequent downregulation of CD24 was confirmed by both qRT-PCR and western blotting (**Figures S6A–C**). SCC084/EV and SCC084/miR-146a cells were then introduced in the right and left flanks of NOD/SCID mice respectively and allowed to form subcutaneous tumor (**Figures S6D, E**). It is interesting to note that in stable lines, there is an enhancement in β-catenin mRNA level, which is most likely due to prolonged miR-146a expression that may have potentiated a stable CSC phenotype (**Figure S6B**) (20, 39). A significant increase in tumor volume and tumor weight was observed in SCC084 xenografts stably over-expressing miR-146a suggesting enhanced tumorigenic potential of these cells (**Figures 7B, C**). Further, to explore the effect of AKT signaling on miR-146a induced tumor, mice with palpable tumors generated from SCC084/EV and SCC084/miR-146a cells were treated with quercetin, a known PI3K/AKT signaling pathway blocker (**Figures S6D, E**) (40). As expected, the tumor formation ability of miR-146a cells was significantly attenuated *in vivo* upon administration of quercetin at regular intervals (**Figures 7B, C**). While there was no effect of quercetin on miR-146a and CD24 levels, β-catenin over-expression in miR-146a tumors was compromised in presence of quercetin (**Figures S6F, G**). In order to investigate the effect of CD24 upon the acquired tumorigenicity of miR-146a expressing cancer cells *in vivo*, we examined xenograft tumors generated from SCC084/miR-146a cells harboring CD24 expression construct (**Figures S6A–C, H, I**). Notably, compared to the control tumors, volume and weight of CD24 expressing SCC084/miR-146a tumors were not significantly (p-value = 0.7360) altered (**Figures 7E, F**) suggesting loss of miR-146a driven tumor formation ability. Xenograft tumor subjected to qRT-PCR analysis confirms the overexpression of miR-146a and CD24 in these cells while β-catenin is down-regulated (**Figures S6J, K**). Immunohistochemical analyses further confirmed the increased β-catenin protein levels in miR-146a expressing tumor specimens which is diminished upon either quercetin treatment or CD24 over-expression (**Figures 7D, G**). Collectively, these results indicate that CD24/AKT/β-catenin axis plays an important role in miR-146a mediated tumor growth *in-vivo*.

### β-Catenin Transactivates miR-146a Expression Contributing to Positive Feedback Loop

**Figure 8:**
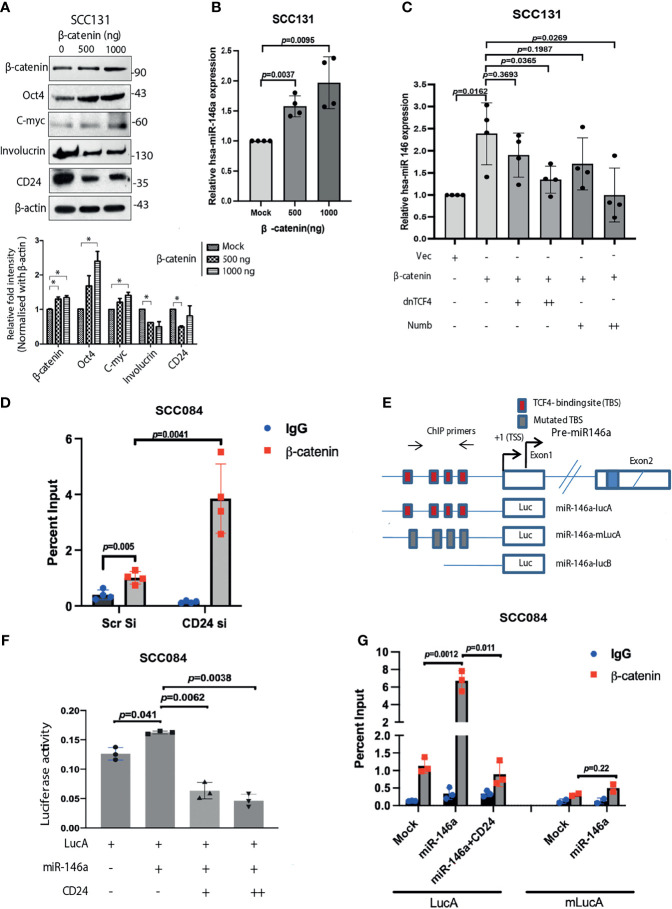
β-catenin transactivates miR-146a mediating a positive feedback loop. **(A)** Increase in stemness markers upon β-catenin over-expression as revealed by western blot and the densitometric analysis of its band intensities (right below). Data was normalized with corresponding β-actin (*P < 0.05). **(B)** Concomitant fold change of miR-146a expression in β-catenin transfected SCC131 as quantified by qPCR. **(C)** Increase in miR-146a transcripts upon β-catenin over-expression is dose dependently inhibited in the presence of either dnTCF4 or Numb. Unpaired student’s t-test were performed on log transformed fold change values in GraphPad prism 8. **(D)** Chromatin Immunoprecipitation assays in SCC084 cells transfected with either scramble siRNA or CD24 siRNA showing recruitment of β-catenin upon endogenous miR-146a promoter. We used unpaired t-tests on %input to calculate *p-*values. **(E)** Schematic of miR-146a promoter locus and reporter constructs namely LucA: wild-type; mLucA: TCF-4 binding site (TBS) mutation; LucB: TBS deletion (20). **(F)** Relative luciferase activity of LucA in SCC084 cells under various transfections as indicated (one sample t-test was used to compute p-values). Data represent average of n = 3 individual experiments. **(G)** Transient ChIP assay with same constructs as shown. The percentage enrichment of amplified product was normalized to input and graphically presented. Data represent mean ± sd and n = 3 different experiments. We used unpaired t-tests calculate *p-*values.

The upstream regulators of miRNAs have always been involved in feedback regulatory mechanisms and are not much investigated. Analysis of miR-146a promoter has revealed the binding sites for *NF-κB, TCF4/β-catenin* and *STAT3*, suggesting possible transcriptional modulation (20, 41, 42). Our data suggests that β-catenin directly enhance stemness features by driving the intracellular levels of *c-Myc* and Oct4 (**Figure 8A**). This apparently contributes to the enhanced tumorigenic properties which was observed in response to high miR-146a levels. Interestingly, we found that β-catenin in turn also promotes the expression of miR-146a, which might augment the stemness acquiring ability of the cancer cells (**Figure 8B**). However, expression of miR-146a was significantly reduced in the presence of both dn*TCF4* which inhibits β-catenin binding to the promoter and numb which degrades it respectively (**Figure 8C**). Change in miR-146a promoter activity under similar conditions suggests that β-catenin is involved in trans-activation of miR-146a promoter (**Figure S7A**). We hypothesize that β-catenin mediated induction of miR-146a contributes to β-catenin mediated CD24 reduction as shown earlier (**Figure 8A**). ChIP-qPCR assay using β-catenin antibody confirmed that β-catenin binds to the miR-146a promoter *in vivo* (**Figure 8D**). Further, the recruitment of β-catenin was found to be significantly enhanced upon knockdown of CD24 suggesting negative regulation of β-catenin by CD24 (**Figure 8D**). Moreover, miR-146a promoter activity was significantly increased in presence of miR-146a, while reduced upon ectopic expression of CD24 (**Figures 8E, F**). However, the constructs with either mutated (m-LucA) or deleted (LucB) TCF4 binding sites showed reduced difference in promoter activity in presence of miR-146a (**Figure S7B**). This may be due to the alternating levels of β-catenin which shoots up in miR-146a over-expression condition and gets depleted in presence of CD24. Transient ChIP assays with the same luciferase constructs also confirmed that β-catenin indeed binds to miR-146a promoter, which is impeded upon CD24 over-expression (**Figure 8G**). These data positively confirm the feedback activation loop by β-catenin that further trans-activates miR-146a expression to shift the equilibrium towards CSC maintenance.

## Discussion

Oral cancer progression has been largely attributed to both genetic and epigenetic alterations of the cellular genome. Tumorigenic cells can arise from the non-tumorigenic cancer cells owing to spontaneous conversion to a stem-like state (43). The origin and plasticity of such cells, called cancer stem cells (CSCs), have always been a matter of debate and is most likely attributed to transient molecular changes. Nevertheless, CSCs are suspected to be responsible for the underlying chemo-resistance, recurrence and metastasis of a tumor (34). Detail molecular characterization of CSCs is therefore a pre-requisite for identifying and eliminating them from its roots. However, no single molecular marker can exclusively be assigned to distinguish CSCs from other cancer cell types. CD24 has been routinely used in combination with CD44 for the prospective isolation of CSCs in colorectal, prostate and breast cancers (34, 44). Expression of Aldehyde dehydrogenase, drug transporters like *ABCG2* and various other signaling molecules are also known to mark CSCs in a given tumor (32). It is assumed that the cellular miRNAs regulating these CSC markers may provide an important target for anticancer therapy (19, 45). For instance, targeting of CD44 by miRNAs in NSCLC, prostate and ovarian cancer has been demonstrated to attenuate stemness (46). However, miRNA mediated regulation of CD24 remains to be determined.

Consistent with its oncogenic functions, miR-146a promotes symmetric division of colorectal CSCs, thereby promoting stemness (20). This miRNA is also involved in the development of melanoma by activating Notch1 signaling leading to drug resistance (47). However, little is known about its role in regulating expression of CSC-related CD markers in oral cancer. In the present study, we provide evidence that miR-146a mediated downregulation of CD24 confers CSC phenotype in oral cancers. We also show that decreased level of CD24 leads to stabilization of β-catenin due to degradation of AKT. Firstly, we demonstrated significantly higher expression of miR-146a in CD44^high^CD24^low^ population of OSCC cell lines as well as in tumor specimens. We therefore investigated whether miR-146a expression maintains CSC traits or miR-146a accumulation is a consequence of induced stemness. Notably, ectopic expression of miR-146a induced enrichment of CD44^high^CD24^low^ population together with increased β-catenin activity in OSCC cell lines. The molecular connection for miR-146a induced CD44 expression was understandable as CD44 is a well-known transcriptional target of β-catenin (20). However, the effect of miR-146a upon CD24 expression under these conditions was particularly unknown. Hence, we examined whether CD24 is a direct target of miR-146a and experimentally confirmed that miR-146a binds to the 3’UTR of CD24 thereby repressing it post-transcriptionally. This observation was quite intriguing as direct targeting of cell surface CSC markers by miRNA could be therapeutically beneficial. However, we cannot rule out that there are alternative targets of mir-146a, which could also possibly contribute synergistically to the effect in oral cancer stemness. Besides, miR-146a is known to be expressed in normal tissue with some relevant physiological roles that may be hindered (48). This adds to the potential limitations for using miR-146a in therapy and needs further investigation. From this study, loss of E-cadherin upon miR-146a over-expression and positive correlation with the mesenchymal marker vimentin was also evident. Hence, in addition to its novel role in acquiring stemness, our results also confirmed miR-146a as a key regulator of EMT (49).

Wnt/β-catenin signaling has shown great potential for CSC-targeting in cancer (33). Our study shows that CSC characteristics in OSCC is attributed to the elevated β-catenin along with depleted CD24 levels. The anticipation that CD24 leads to proteasomal degradation of β-catenin was found to be true and apparently it also abolished the β-catenin mediated stemness. This is a novel functional interaction through which miR-146a regulates β-catenin in oral cancer cells. Nuclear localization of β-catenin in stable miR-146a expressing tumor was evident, however, less pronounced upon transient expression in cell lines, which likely indicates the equilibrium shift towards CSCs, due to prolonged expression of miR-146a with consequent reduction of CD24. Our study, thus points towards the tumor-suppressor functions of CD24, supporting our previous observation of reduced CD24 expression in oral tumors compared to the normal tissue (2). Although growth inhibition was achieved by knocking down CD24 in colorectal and pancreatic cancer, no such effects were observed in oral cancer. Perhaps the variable cell-type specific effect underlies the paradoxical role of CD24 in oral cancer (50).

Activated PI3K-AKT pathway is one of the primary events in carcinogenesis (51). Its contribution to stem cell self-renewal and proliferation has also been extensively studied. Receptor Tyrosine Kinase (RTKs) mediated growth signals (through EGF, IL-6, TGF-β etc.) impinges upon AKT through activation of the PI3K kinase. This RTK mediated activation of AKT *via* PI3K is also negatively regulated by PTEN which again is known to be regulated by miR-21 (52, 53). Stability of phospho-AKT and other kinases play key role in maintaining its activity in cancer stem cells of chronic myeloid leukemia (CML), NSCLC, breast, prostate and colorectal cancer (54). Further, signaling pathways like WNT are often linked with AKT activation that eventually contribute to expression of stem cells-related factors, chemo-resistance genes, and CSC markers (51, 55). Here we show that CD24, the cell surface CSC marker lie upstream of AKT protein, along with TWIST and FOXO transcription factors which in turn is also known to inhibit CD24 (56). However, the precise mechanism by which expression/stability of AKT protein is regulated by CD24 is still unknown. CD24 has been shown to possibly modulate phospho-AKT levels (57), which might affect its downstream targets such as GSK-3β (37). Activated GSK-3β mediates phosphorylation and ubiquitination of β-catenin, thereby leading to its degradation (37). Therefore, it was incumbent on us to ask whether CD24 mediates inhibition of AKT and subsequently affect β-catenin stability in oral CSCs. Indeed, MG132 treatment was found to re-stabilize β-catenin by relieving pAKT inhibition in cells over-expressing CD24. Moreover, direct AKT inhibition in miR-146a expressing cells depleted β-catenin, irrespective of CD24 level suggesting AKT is downstream of CD24. Thus, we logically elucidated the molecular mechanism underlying CD24 mediated β-catenin degradation *via* AKT in oral cancer cells.

Our findings thus not only supported the importance of signaling molecules in CSC maintenance, but also elucidated an upstream regulatory network that may be broadly applicable for other pathways. We have specifically shown that CD24 over-expression decrease levels of phospho-AKT leading to β-catenin instability. The role of miR-146a/CD24/AKT/β-catenin axis in maintaining the oral cancer stem cell populations is thus mechanistically evident. Studies from in-vivo tumor model system also confirms that this molecular mechanism directly affects tumorigenesis in OSCC. Further, the recruitment of β-catenin onto the miR-146a promoter was found to be negatively regulated by CD24 which might contribute to the fine tuning of stemness. These results clearly establish a cross-regulatory network between miR-146a and β-catenin, governed by a stem-related marker, CD24 in OSCC cells.

## Conclusions

Our study thus provides strong evidence which suggest that miR-146a promotes CSC characteristics of oral cancer cells by down-regulating CD24. Repression of CD24 leads to AKT stabilization followed by activation of Wnt/β-catenin signaling. Based on our observation, we propose a model wherein, AKT activity is an important determinant of miR-146a dependent β-catenin signaling (Graphical abstract). It should be noted, however, that β-catenin mediated CSC induction might be due to the induction of miR-146a expression or vice-versa. Taken together, the present study highlights a novel mechanism of miR-146a mediated self-renewal capacity of Oral CSCs that may have a prognostic or therapeutic value in oral cancer.

## Data Availability Statement

The original contributions presented in the study are included in the article/**Supplementary Material**. Further inquiries can be directed to the corresponding author.

## Ethics Statement

The animal study was reviewed and approved by National Centre for Cell Science, Pune.

## Author Contributions

SG and SuR conceived and designed the study. Some experiments were designed by DG and SD. Experiments, data collection and statistical analyses were performed by SG, DG, SD, StR and PD. Some experiments were performed by RB and MG. The manuscript was written and edited by SG, DG, SD, SN, GCK, and SuR. All authors contributed to the article and approved the submitted version.

## Funding

The work was supported by CSIR --Mayo Clinic Collaboration for Innovation and Translational Research Grant CMPP-08 and J.C. Bose National Fellowship grant JCB/2017/000005 awarded to SuR. SN is supported by Early Career Award, Science & Engineering Research Board (SERB)-Dept. of Science and Technology (DST), Govt of India (File No. ECR/2015/000206) and Grant-in-Aid, Department of Science & Technology and Biotechnology, Govt. of West Bengal (ST/P/S&T/9G-21/2016). SG, DG and PD are supported by fellowship from the Council of Scientific and Industrial Research (New Delhi, India). SD and RB are supported by Dr. Shyama Prasad Mukherjee Senior Research Fellowship (SPM-SRF) and Senior Research Fellowship from CSIR respectively. StR is supported by University Grants Commission- Junior Research Fellowship (UGC Ref. No.:771/ CSIR-UGC NET-2017).

## Conflict of Interest

The authors declare that the research was conducted in the absence of any commercial or financial relationships that could be construed as a potential conflict of interest.

## Publisher’s Note

All claims expressed in this article are solely those of the authors and do not necessarily represent those of their affiliated organizations, or those of the publisher, the editors and the reviewers. Any product that may be evaluated in this article, or claim that may be made by its manufacturer, is not guaranteed or endorsed by the publisher.

## Supplementary Material

Supplementary File 1 List of Primers for the qRT-PCR (Excel File)

Supplementary File 2 Supplementary Figure and Figure Legends (S1, S2, S3, S4, S5, S6, S7)

Supplementary File 3 Analysis of TCGA data for Head and Neck Squamous Cell Carcinoma patients (Excel File)

Supplementary File 4 Snapshots of miRanda analysis for miR-146a-5p seed sequence and CD24 3’UTR matching showing their free energy of binding (pdf)

## Abbreviations

OSCC, oral squamous cell carcinoma; CSCs, cancer stem cells; mi-RNAs, micro-RNAs; HNSCC, Head and Neck Squamous cell carcinoma.
