# Supplementary material for: MiRNA-146a/AKT/β-Catenin Activation Regulates Cancer Stem Cell Phenotype in Oral Squamous Cell Carcinoma by Targeting CD24
Source: Front Oncol. 2021 Oct 12;11:651692. doi: 10.3389/fonc.2021.651692 (PMC8546321; doi:10.3389/fonc.2021.651692)
Supplement: Supplementary File 1 — List of Primers for the qRT-PCR (Excel File) [file DataSheet_1.zip › Supplementary File 2.pdf]

Supplementary Information: Supplementary File 2

Ghuwalewala et al. MiR-146a dependent regulation of CD24/AKT/β-catenin axis drives cancer stem cell phenotype in oral squamous cell carcinoma.

Supplementary File 2, Table 1: The characteristics and phenotype of the SCC cell lines established from HNSCC tumour specimens:

All three UPCI: SCC cell lines* used in this study had 11q13 amplification and a wild type p53, whereas SCC25 was a mutant p53 cell line obtained from ATCC.

Cell line	Characteristics	Phenotype
UPCI: SCC131	Derived from the T2N2, well differentiated tumor in the floor of mouth (male age:56)	Moderate expression of miR146a, CD44 and CD24, that correlated well with its tumorigenic behaviour
UPCI: SCC084	Derived from the T2N2B, moderately differentiated tumor in the retromolar trigone (male age:52)	Expression of CD44 was higher while CD24 was lower than SCC131, that justifies its higher proliferative ability in adherent conditions. However miR-146a levels were not as high, suggesting some alternative means of regulation in this cell line.
UPCI: SCC036	Derived from the T3N1, moderately differentiated tumor in the tonsil (male age:73)	Behaved similar to SCC131, albeit with slightly lower tumorigenicity (lesser colony forming ability in soft agar assay; data not shown)
ATCC:SCC25	Derived from the well differentiated tumor in the tongue (male age:70)	Capable of limited growth in tumorigenic assays, having moderate levels of miR146a, CD44 and CD24

* (White et al., 2007)

Supplementary File 2, Table 2: Multiple correction analysis for various miRNAs as shown in Figure 1a.

	p-value	holm	hochberg	hommel	bonferroni	BH	BY	fdr	none
hsa_miR_125b	0.04321018	0.21605088	0.141049	0.10578675	0.38889158	0.06843172	0.19359116	0.06843172	0.04321018
hsa_miR_100	0.25970454	0.25970454	0.25970454	0.25970454	1	0.25970454	0.7346959	0.25970454	0.25970454
hsa_miR_200b	0.05189059	0.21605088	0.141049	0.10578675	0.46701531	0.06843172	0.19359116	0.06843172	0.05189059
hsa_miR_138	0.00282376	0.01976635	0.01976635	0.01976635	0.02541387	0.00847129	0.02396501	0.00847129	0.00282376
hsa_miR_34a	0.00106343	0.00850747	0.00850747	0.00850747	0.0095709	0.00478545	0.01353789	0.00478545	0.00106343
hsa_miR_143	0.0705245	0.21605088	0.141049	0.141049	0.63472051	0.07934006	0.22445052	0.07934006	0.0705245
hsa_miR_21	0.02998348	0.1799009	0.141049	0.09403267	0.26985134	0.06746284	0.19085022	0.06746284	0.02998348
hsa_miR_146a	0.00081086	0.00729773	0.00729773	0.00648687	0.00729773	0.00478545	0.01353789	0.00478545	0.00081086
hsa_miR_203a	0.05322467	0.21605088	0.141049	0.10644934	0.47902202	0.06843172	0.19359116	0.06843172	0.05322467

Supplementary Figures and Figure Legends

Supplementary Figure S1

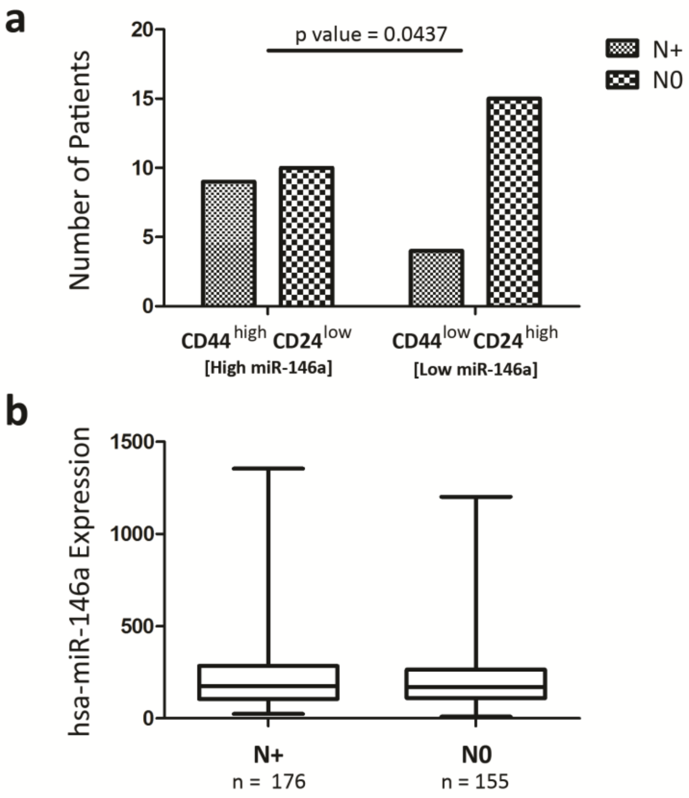

Figure S1. (**a**) Bar graph showing distribution of TCGA HNSCC patients with node positive (N+) and node negative (N0) status within the $CD44^{high}CD24^{low}$ and $CD44^{low}CD24^{high}$ group as classified in Figure. 1c. p-value was calculated using chi-square test. **(b**) Box plots showing the expression of miR-146a (RPM) across the node positive (N+) and node negative (N0) patients from the TCGA HNSCC ("n" represent no. of patients in each group).p-value calculated by unpaired t-test was non-significant.

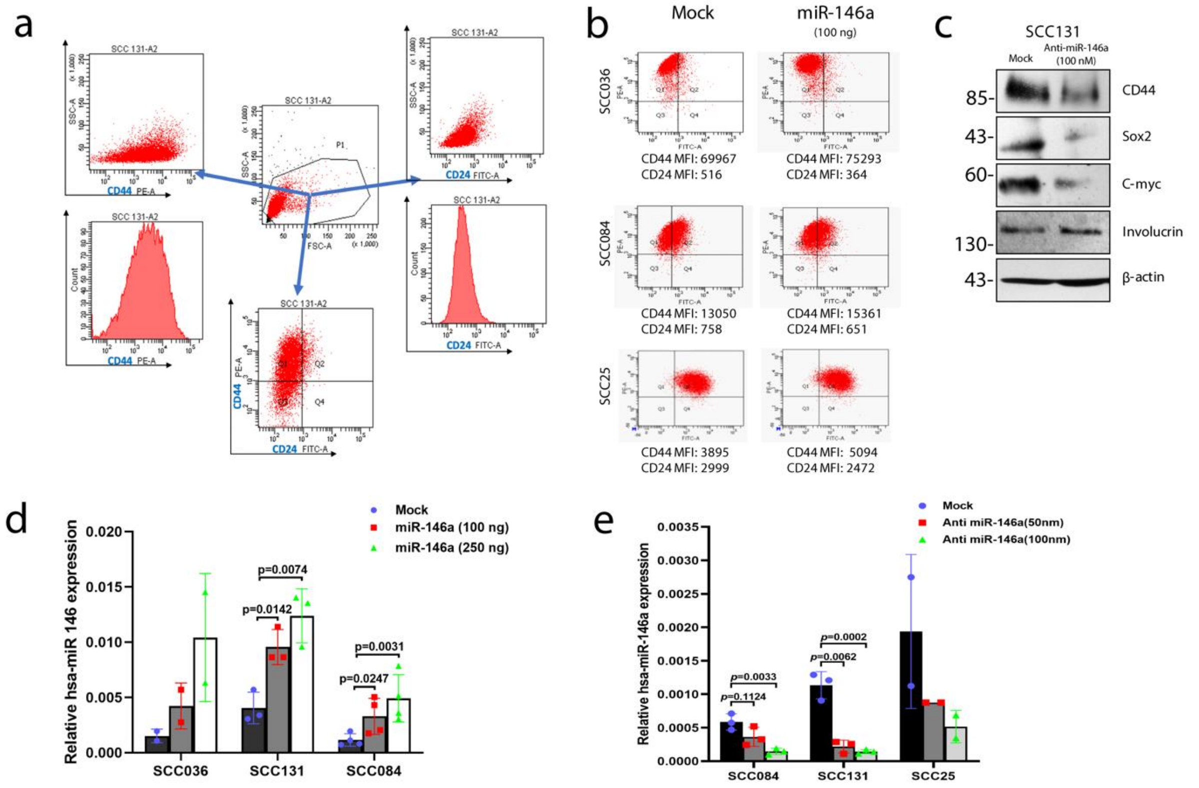

Figure S2. (**a**) Representative gating strategy for the flow cytometry analysis of CD44^{high}CD24^{low} (Q1)and CD44^{low}CD24^{high} (Q4) population. Cells were characterized based on forward and side scatter and mean fluorescence intensity (CD44-PE and CD24-FITC) of the gated populated was directly measured for this experiment. (b) Representative flow cytometry profile of SCC25, SCC084 and SCC036 cell lines with or without miR-146a over-expression stained with CD44 (PE) and CD24 (FITC). Mean fluorescence values are indicated for each cell line. (**c**) Representative Western blot images of CD44, Sox2, C-myc, Involucrin in UPCI: SCC131 transfected with anti-miR-146a. (d) MiRNA specific cDNA derived from SCC036, SCC131, SCC084, and SCC25 upon miR-146a over-expression or (e) knockdown were subjected to qRT-PCR to confirm changes in experimental miR-146a levels. Relative expression values (2^-detCT*100) normalized to those of U6snRNA were plotted. Data shown is average ± sd for three or two independent experiments as indicated by sample dots. Each

experiment refers to biological replicates which were averaged from 3 technical replicates. Unpaired t-test on detCT values was used to compute p-values, respectively shown on the bar graph.

Supplementary Figure S3

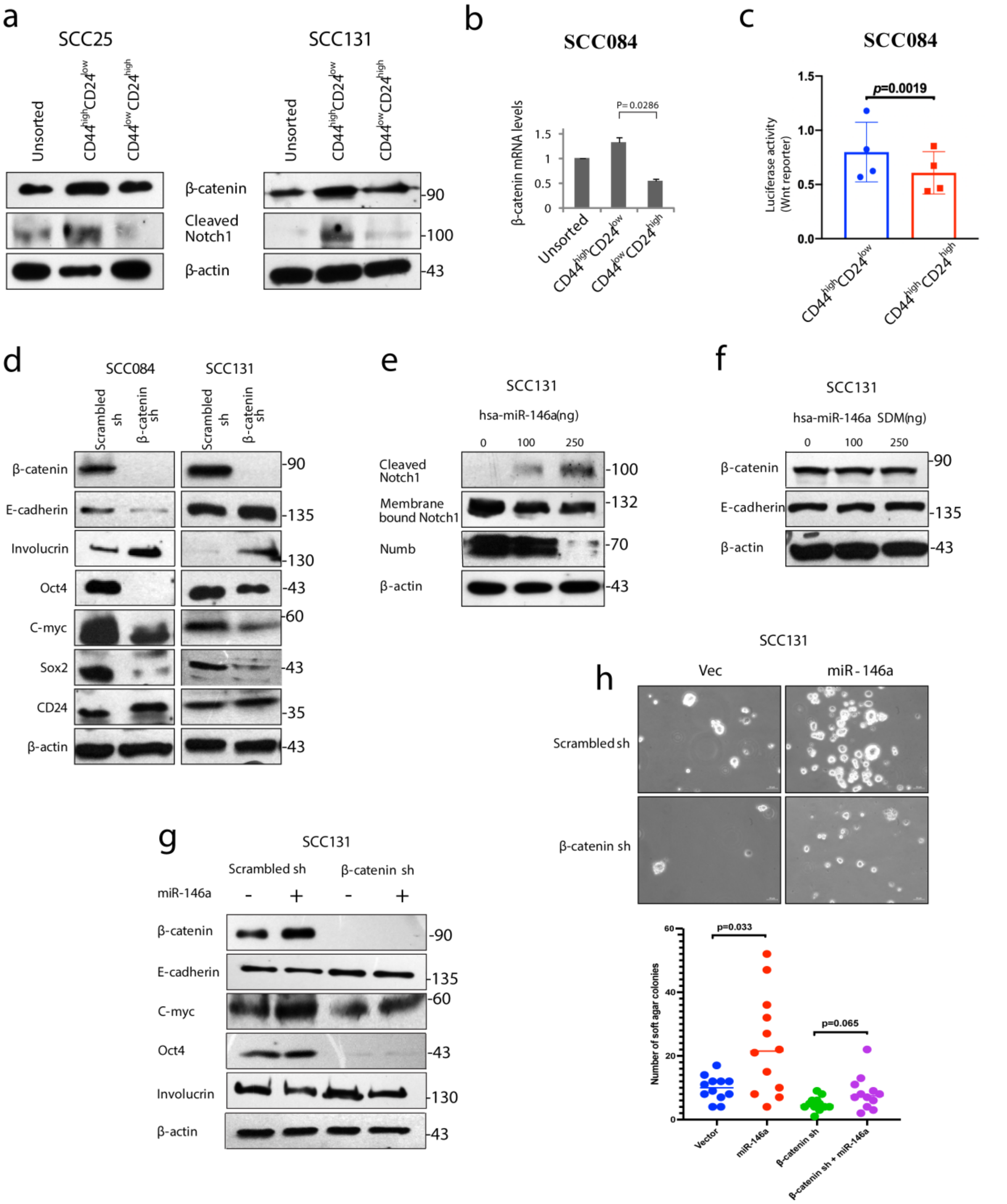

Figure S3. (**a**) Whole cell lysates from $CD44^{high}CD24^{low}$ and $CD44^{low}CD24^{high}$ population of SCC25 and SCC131 were subjected to western blot of Cleaved Notch1 and β-catenin and intensity normalized to β-actin. (**b**) Increased expression of β-catenin in the $CD44^{high}CD24^{low}$ compared to the $CD44^{low}CD24^{high}$population of SCC084 as measured by qRT-PCR (fold change is shown). Data is represented as mean ±sd of at least three independent experiments. (**c**) Relative TOP/FOP luciferase activity in the $CD44^{high}CD24^{low}$ and $CD44^{high}CD24^{high}$ populations. The *P*- value was calculated using one-sample t-test (see methods). (**d**) β-catenin knock-down cells (SCC131 and SCC084 β-catenin sh) along with control (SCC131 and SCC084 scrambled shRNA) cells were lysed and subjected to immunoblotting with stem cell markers. Silencing of β-catenin was also ascertained and β-Actin was used as a loading control. (**e**) Western blots of Cleaved Notch1, membrane bound Notch1 and Numb in miR-146a over-expressing SCC131 cells as a transfection control. (**f**) Immunoblotting of β-catenin and E-cadherin upon miR-146a-SDM transfection. (**g**) Effect of miR-146a transfection on the stemness markers in SCC131 (non-silencing and β-catenin sh) cells by Western blotting. The immunoblot experiment presented is one representative out of two independent experiments. (**h**) Soft agar colonies formed by scrambled and β-catenin shRNA transduced SCC131 with or without miR-146a transfection, followed by their quantification (p value calculated by unpaired Mann Whitney u-test between two independent experiments and each dot represents total number of fields counted). Scale bar=50 µm.

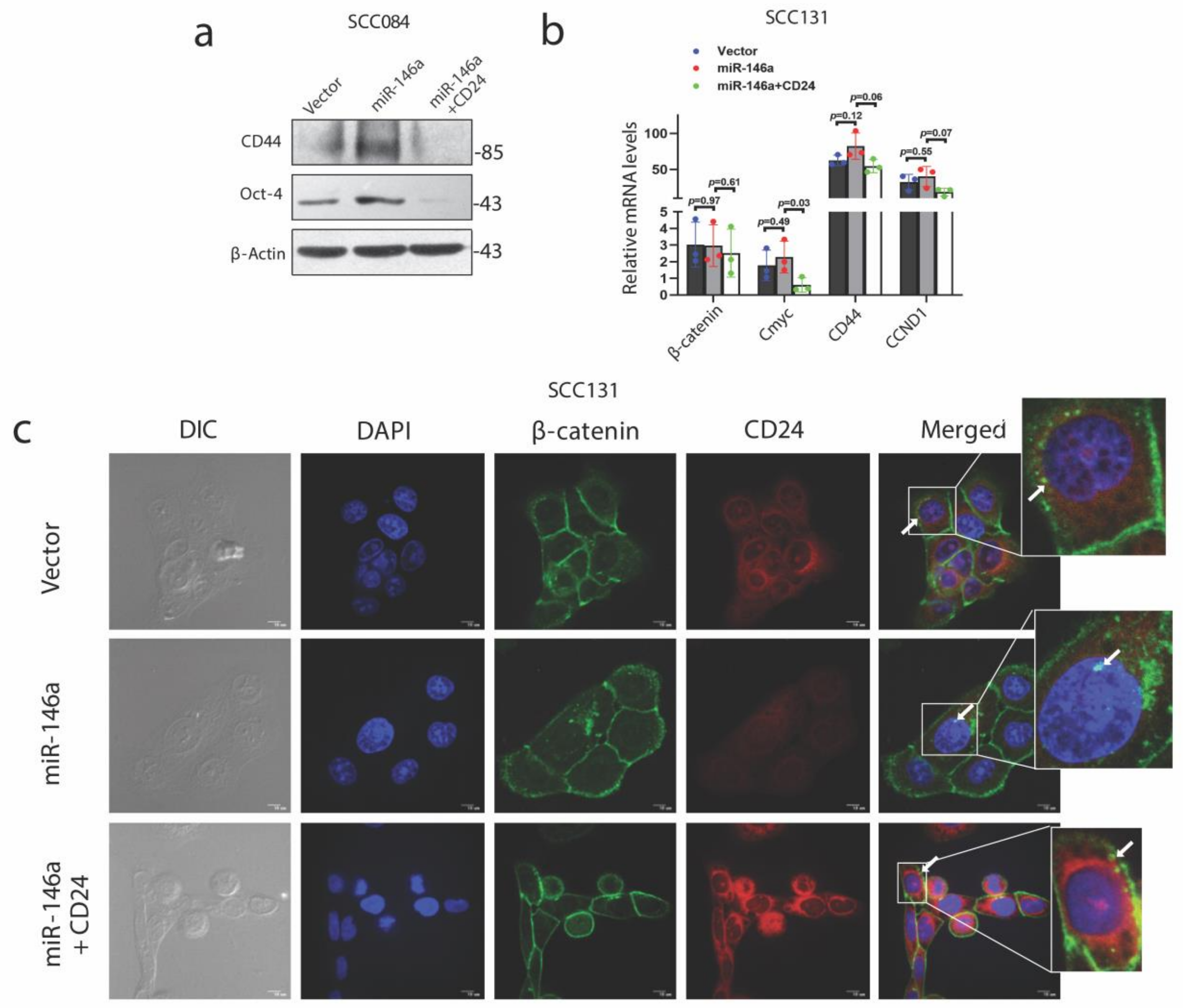

Figure S4. (**a**) Effect of CD24 on CD44 and Oct4 levels in the presence of miR-146a in SCC084 cells. **(b)** Quantification of β-catenin and Wnt target gene (C-myc, CD44 and CCND1) by qRT-PCR transfected with control vector, mir-146a alone or in combination with CD24. Data represent mean ± sd, n=3; n is number of independent experiments. Statistical significance was calculated by unpaired t-test on delCT and *p*-values are shown. (**c**) Representative images of immuno-fluorescent staining of β-catenin and CD24 in SCC131 transfected with a control vector or miR-146a and miR-146a co-expressing CD24 (Red, CD24; green, β-catenin; blue DNA; Scale bars, 10 µm).

Supplementary Figure S5

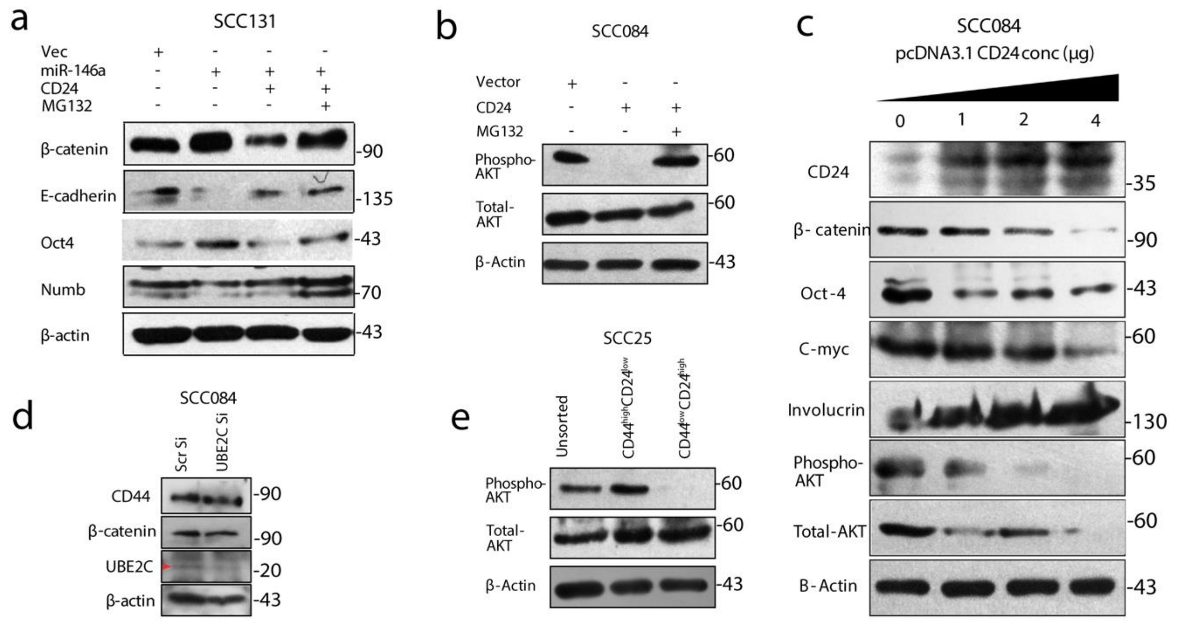

Figure S5. (**a**) Effect of CD24 upon Numb with or without MG132 in SCC131 cells. (**b**) Effect of CD24 upon Phospho-AKT and Total AKT levels in SCC084 cells with or without MG132. (c) Effect of CD24 alone on total Akt/phospho-Akt levels and other stem cell-related proteins (d) Effect of UBE2C siRNA upon CD44 and β-catenin is shown in SCC084 cells (e) Phospho-AKT levels in the CD44^{high}CD24^{low} and the CD44^{low}CD24^{high} subpopulation of SCC25.

Supplementary Figure S6

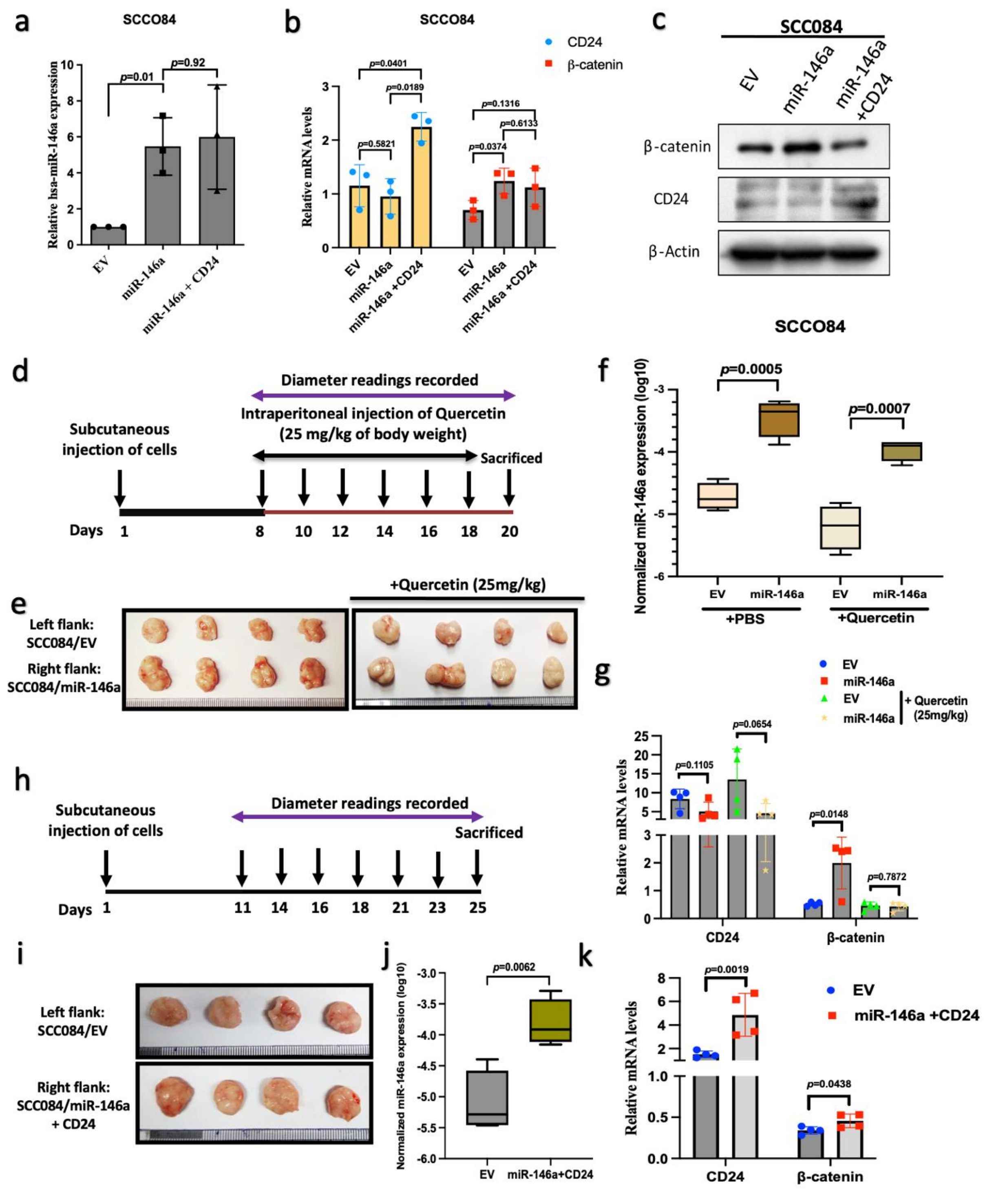

Figure S6. (a) QRT-PCR showing relative expression levels of miR-146a in cells stably expressing either miR-146a alone or miR-146a and CD24.(b) Relative mRNA expression

levels of CD24 and β-catenin in the same conditions as in (a). (c) Immunoblots showing β-catenin and CD24 levels in SCC084 cells as described in (a). (d) Schematic of subcutaneous injection of control or miR-146a expressing cells in the dorsal flanks of mice and timeline of quercetin treatment/recordings of tumor volume. (e) Representative images showing tumor obtained from NOD/SCID mice inoculated with either SCC084/EV or SCC084/146a without or with Quercetin treatment. (f) QRT-PCR data showing relative expression levels (log transformed) of miR-146a in SCC084/EV and SCC084/146a tumors and in response to Quercetin. (g) QRT-PCR data showing relative expression levels of β-catenin and CD24 in SCC084/EV and SCC084/146a tumors in conditions as described in (f). (h) Scheme showing control or miR-146a and CD24 co-expressing cells and various times of tumor measurements. (i) Representative images of tumors generated from SCC084 cells either harboring control empty vector or stably expressing miR-146a and CD24. (j, k) QRT-PCR showing relative expression levels of miR-146a (log transformed) (j), CD24 and β-catenin (k) in tumors as described in (h). β-actin served as the loading control. Bar graphs represent mean SD; n = 4; either delCT or Log transformed values/FCs were subjected to unpaired t-test.

Supplementary Figure S7

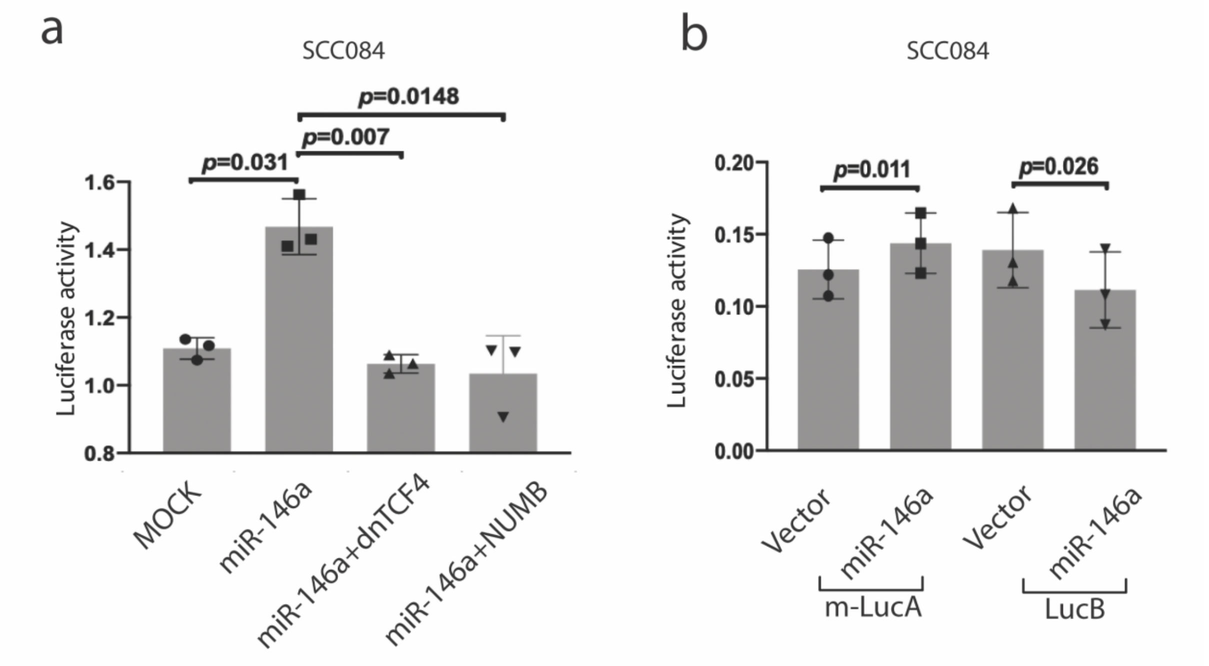

Figure S7. (**a**) LucA activity in the miR-146a over-expressing cells diminished upon dnTCF4 or Numb transfection. (**b**) m-LucA and LucB activity with or without miR-146a in SCC084 cells and relative luciferase activity measured. Data represent mean ± sd, n=3 independent experiments. p values were determined by one-sample t-test.
