# Supplementary figures and images for: MiRNA-146a/AKT/β-Catenin Activation Regulates Cancer Stem Cell Phenotype in Oral Squamous Cell Carcinoma by Targeting CD24
Source: Front Oncol. 2021 Oct 12;11:651692. doi: 10.3389/fonc.2021.651692 (PMC8546321; doi:10.3389/fonc.2021.651692)

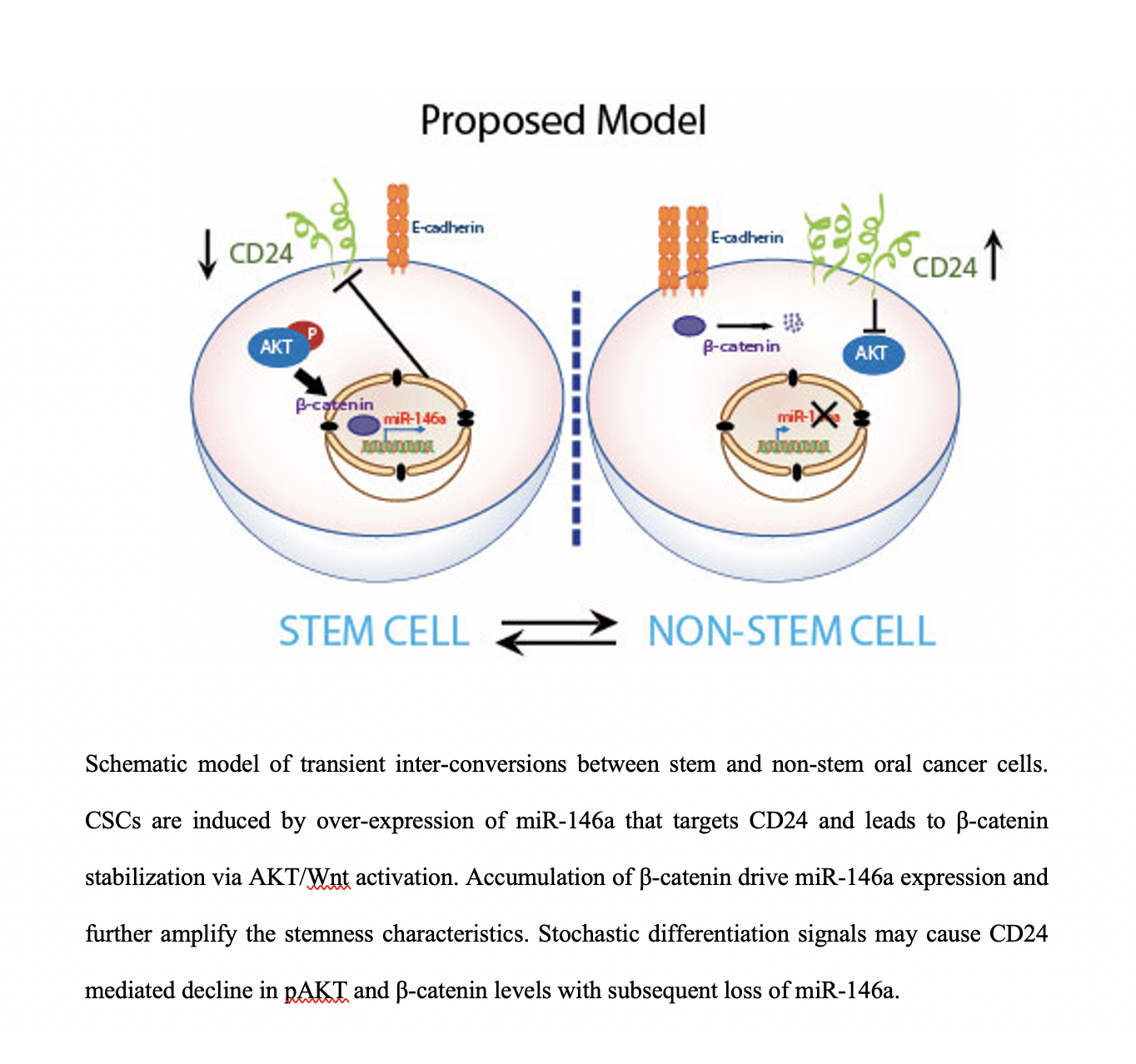

Supplement: Supplementary file 2 [file Image_1.png]
